# Supplementary figures and images for: Systems-level identification of key transcription factors in immune cell specification
Source: PLoS Comput Biol. 2022 Sep 26;18(9):e1010116. doi: 10.1371/journal.pcbi.1010116 (PMC9536753; doi:10.1371/journal.pcbi.1010116)

a

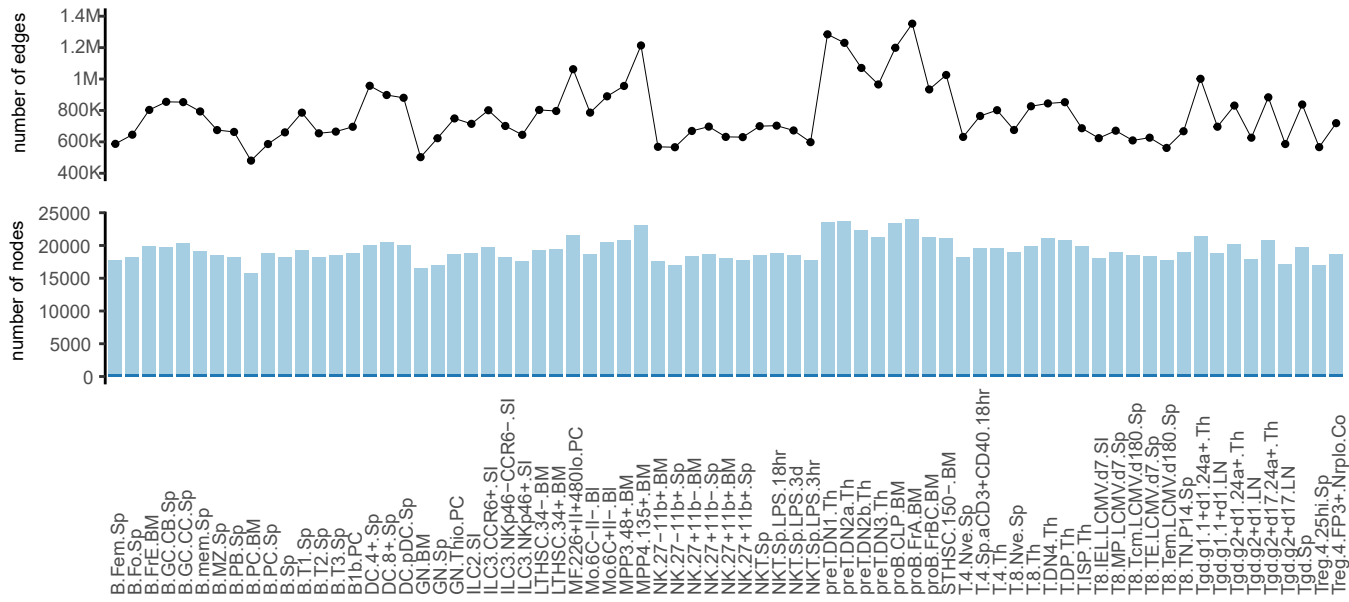

b

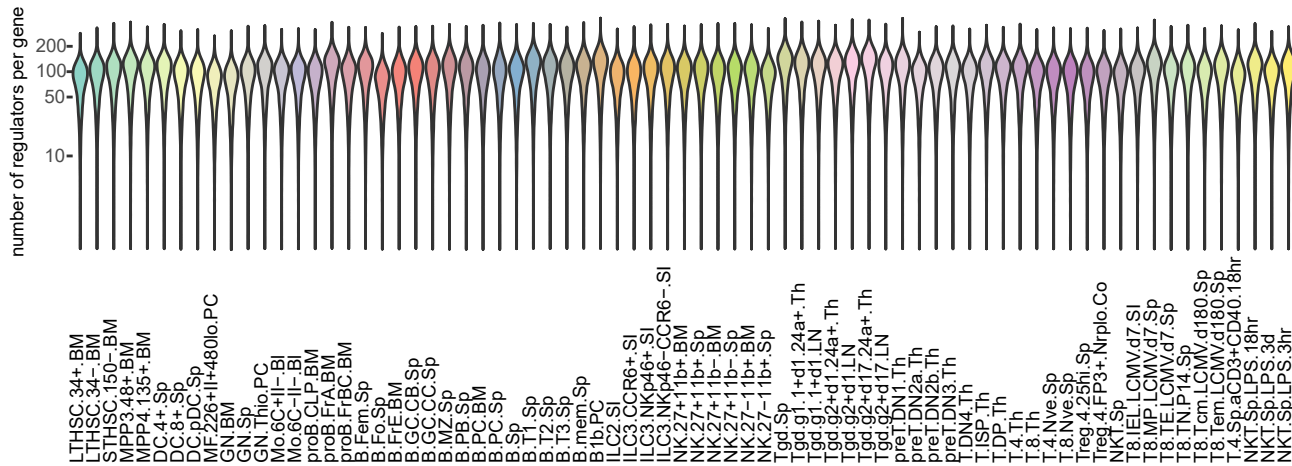

c

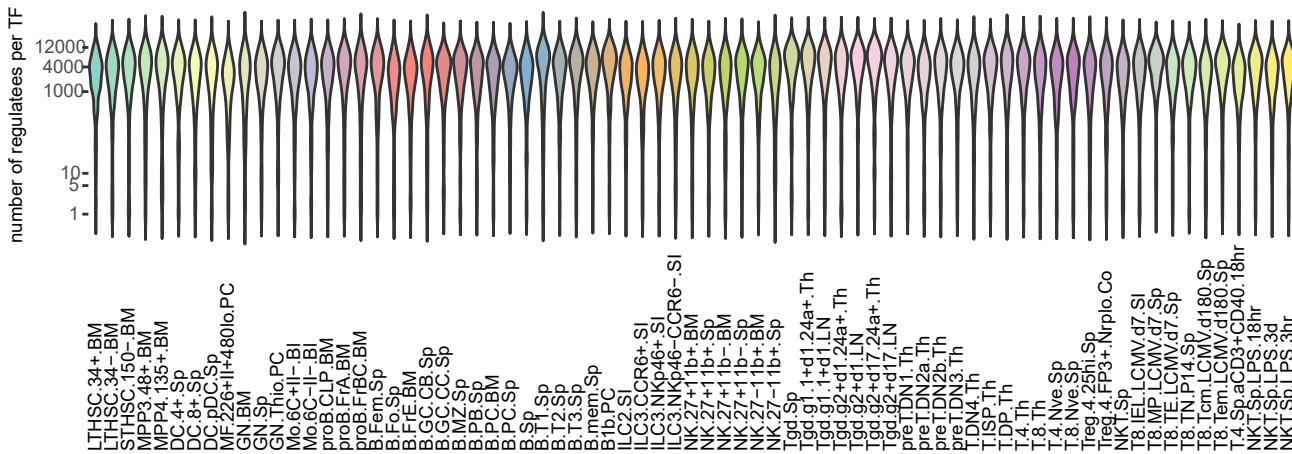

Supplement: S1 Fig — (A) The number of edges (top) and nodes (bottom) in each genetic network. (B) The distribution of the number of regulators per gene for each genetic network. (C) The distribution of the number of regulatees per TF for each genetic network. (PDF) [file pcbi.1010116.s001.pdf]

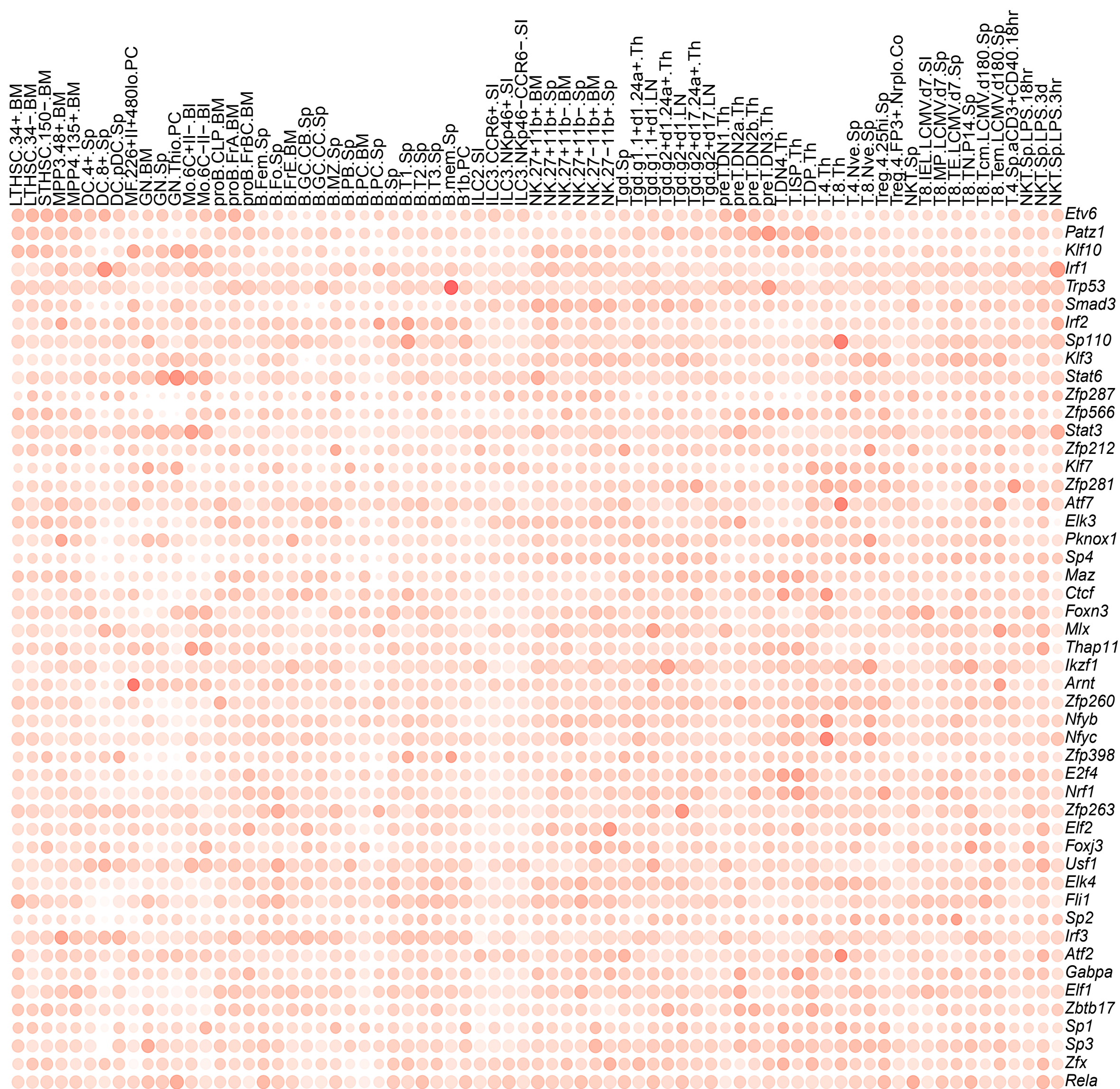

Supplement: S2 Fig — (PDF) [file pcbi.1010116.s002.pdf]

a

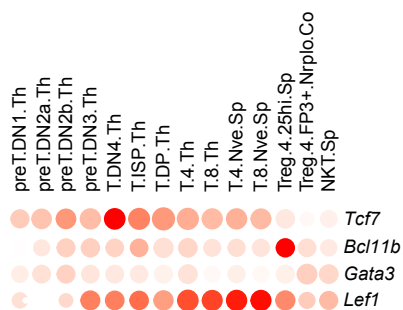

b

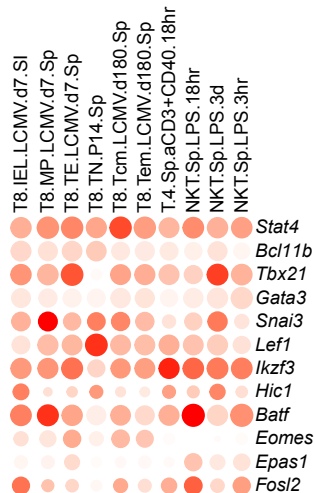

c

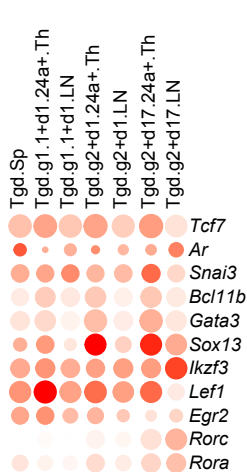

d

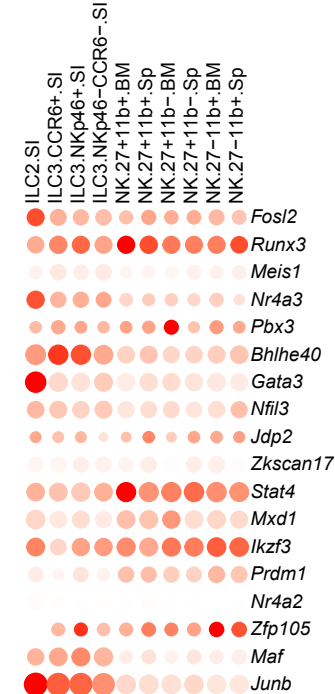

e

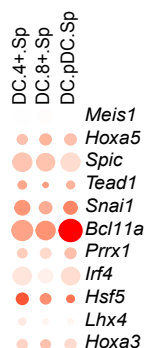

f

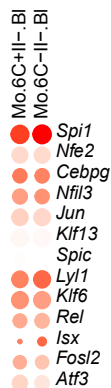

g

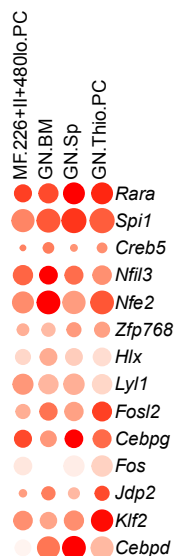

h

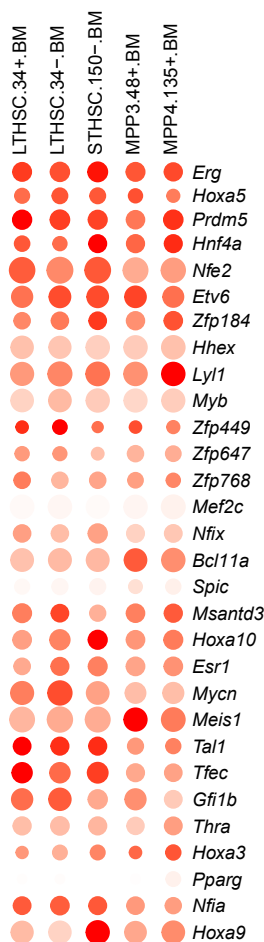

i

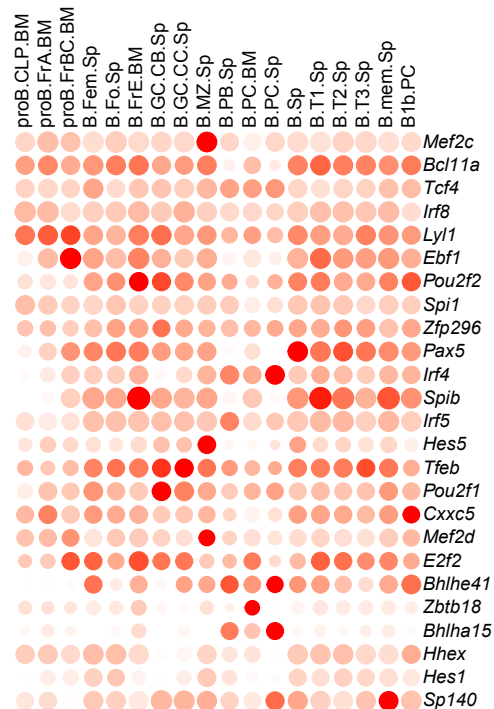

Supplement: S3 Fig — (A) αβT cell. (B) Act_T cell. (C) γδT cell. (D) ILC. (E) DC cell. (F) Mo. (G) MF/GN. (H) Stem cell. (I) B cell. The circle size and color scale are the same as Fig 4. (PDF) [file pcbi.1010116.s003.pdf]

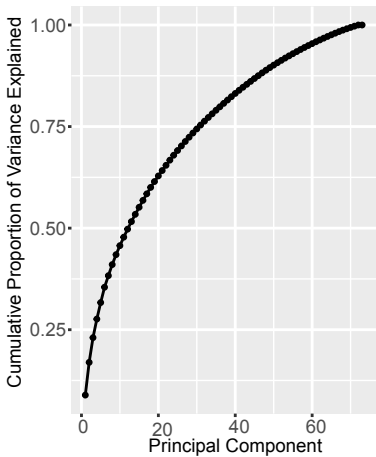

Supplement: S4 Fig — The first 30 PCs were kept according to the “elbow” method. (PDF) [file pcbi.1010116.s004.pdf]

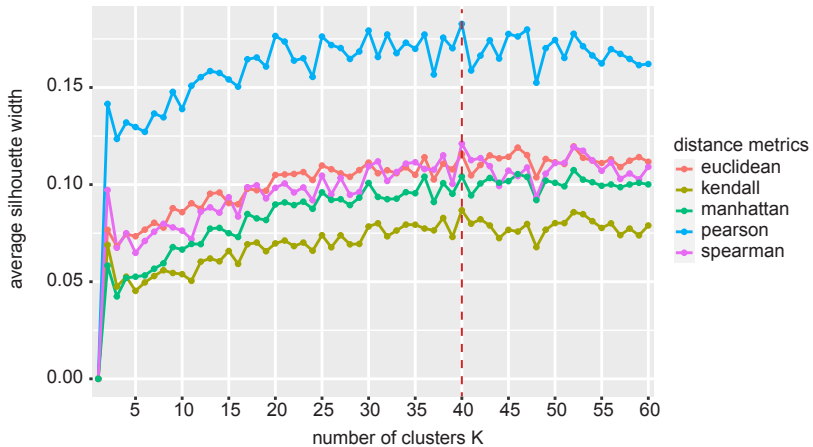

Supplement: S5 Fig — The Pearson correlation was chosen and k = 40 was the ideal cluster number. (PDF) [file pcbi.1010116.s005.pdf]

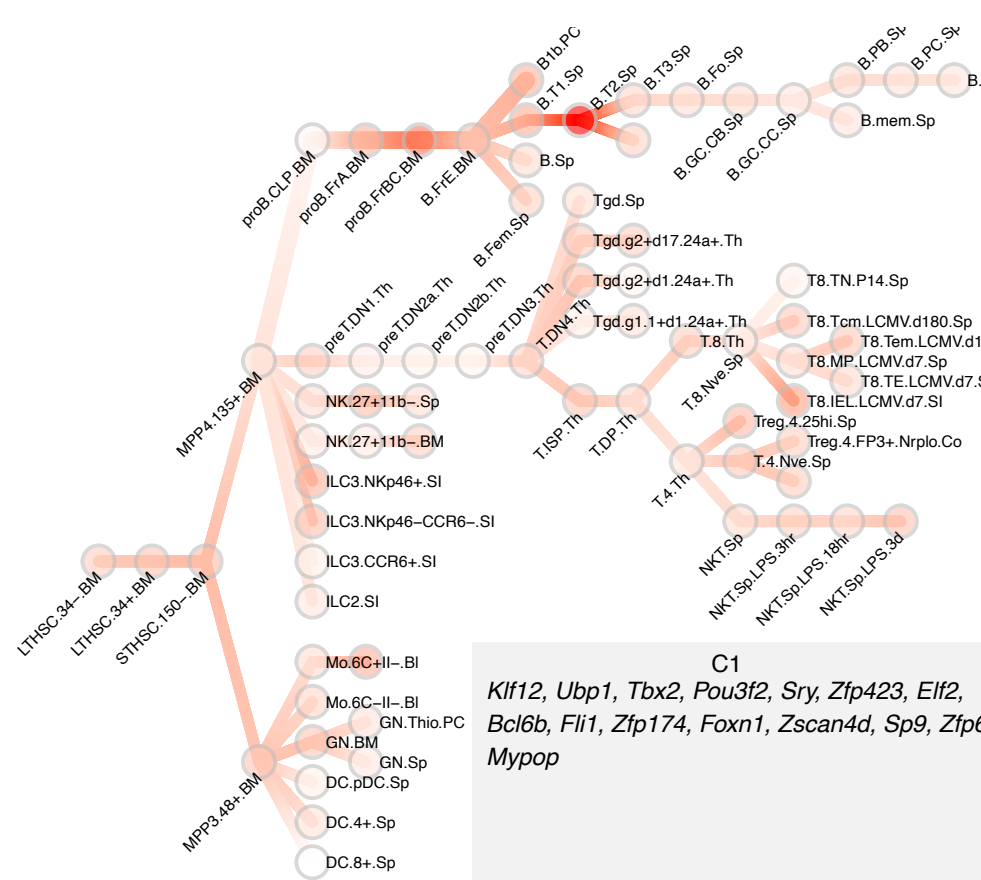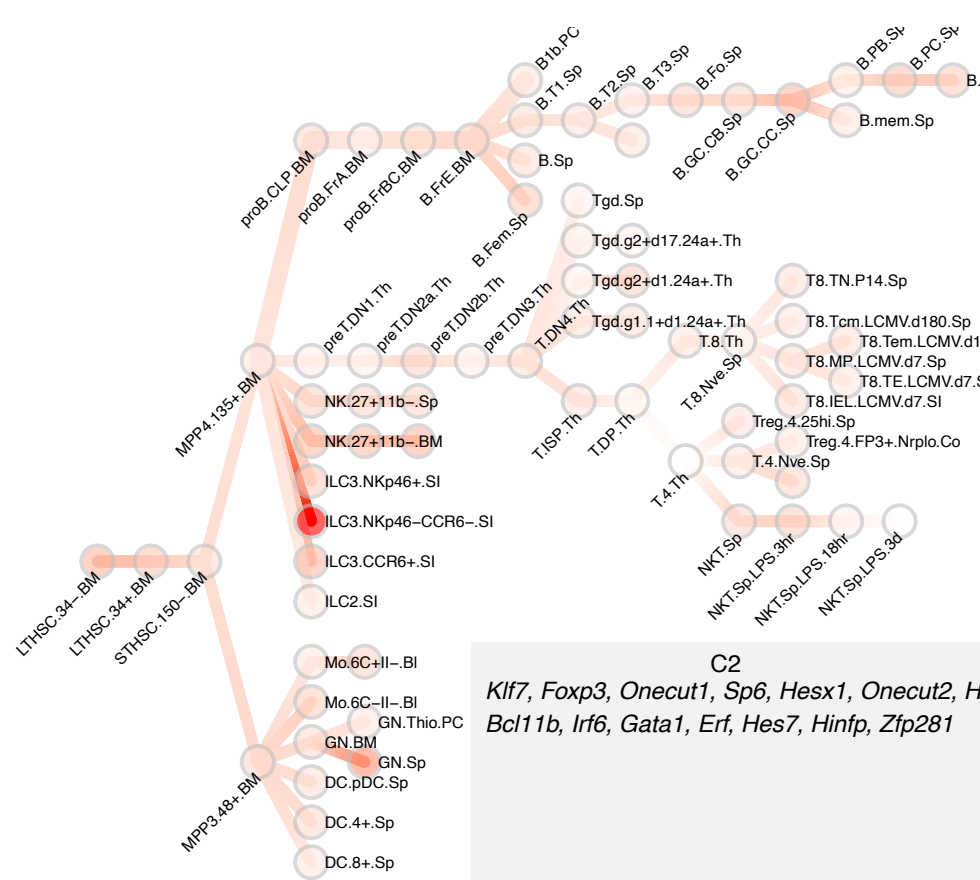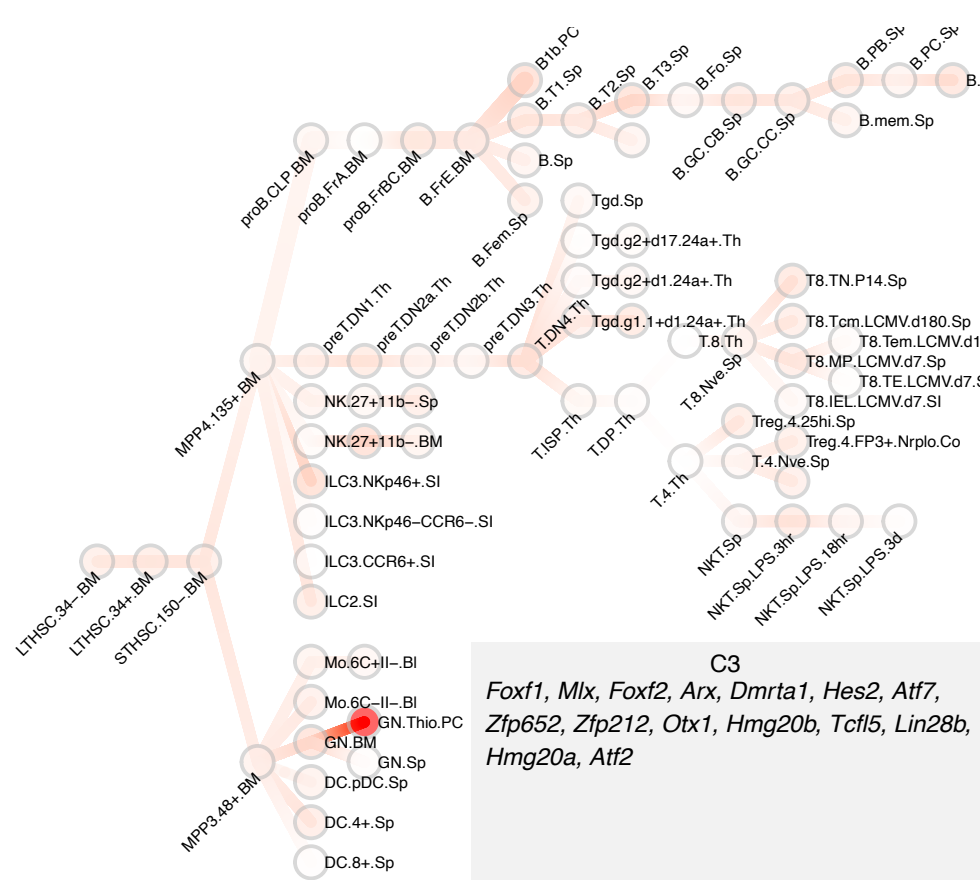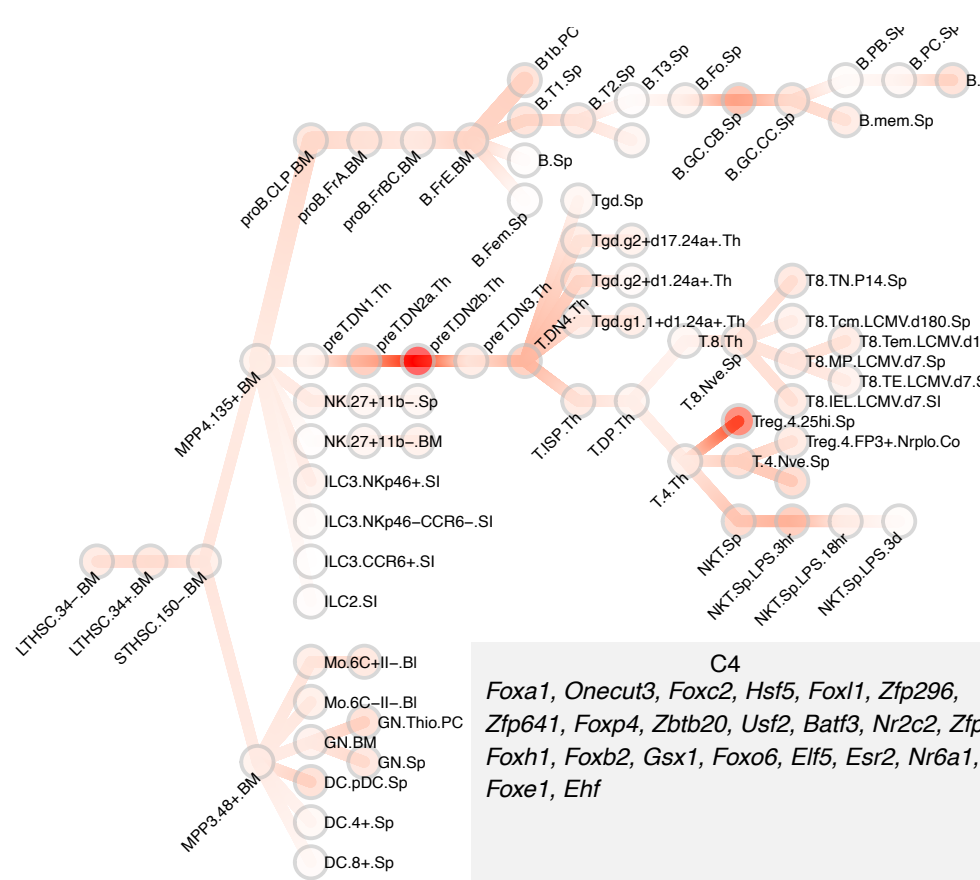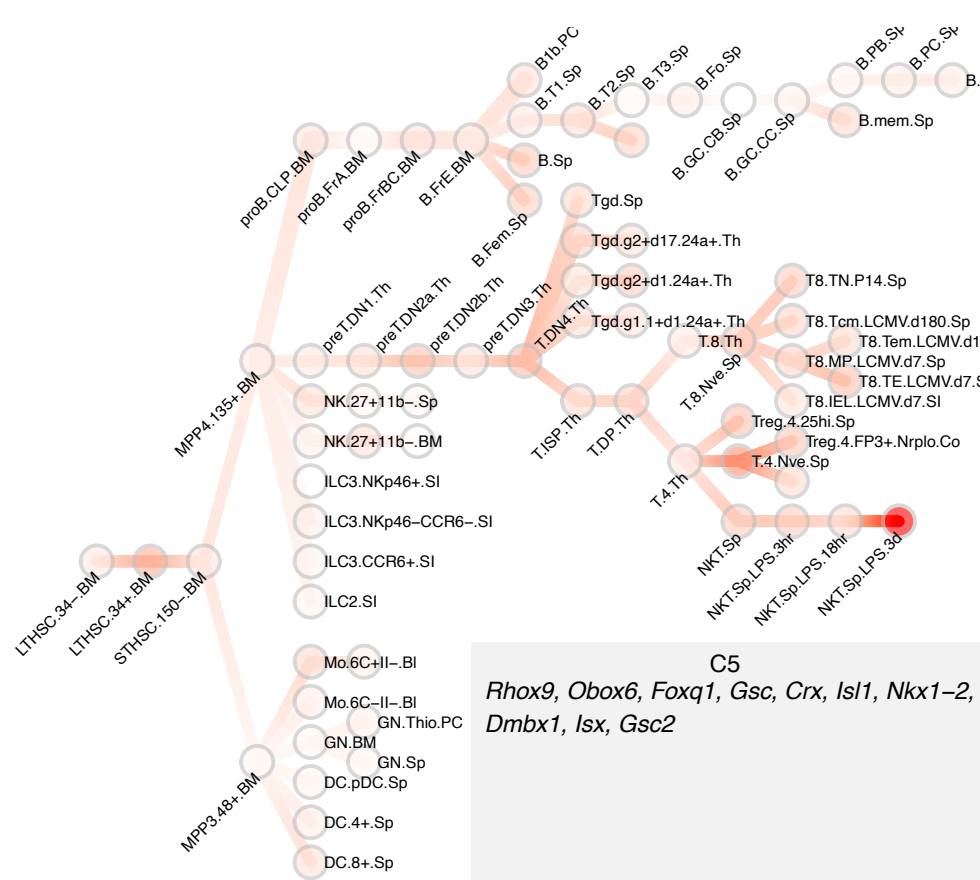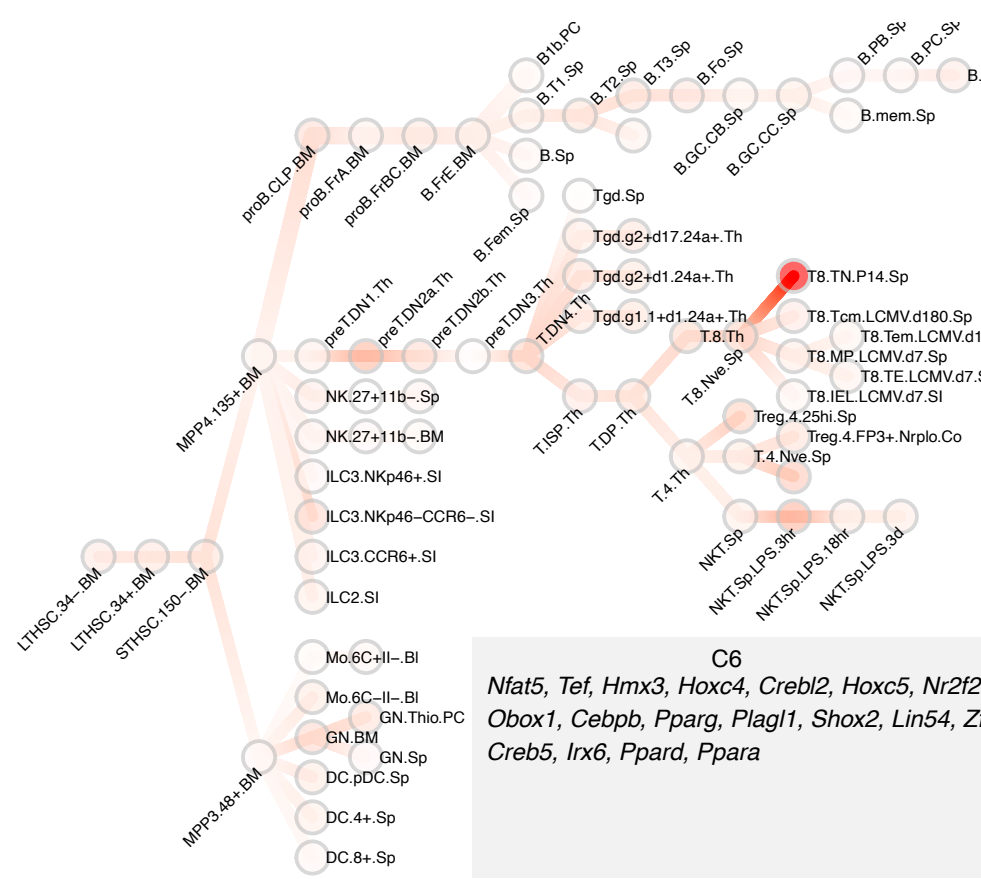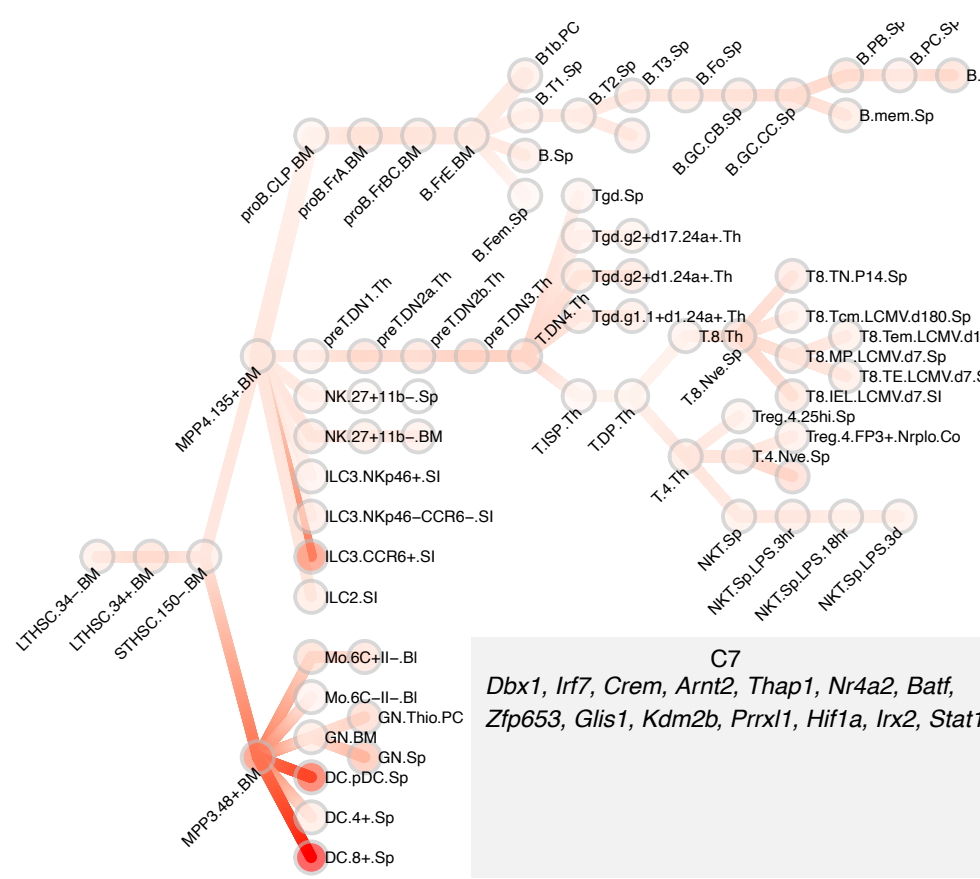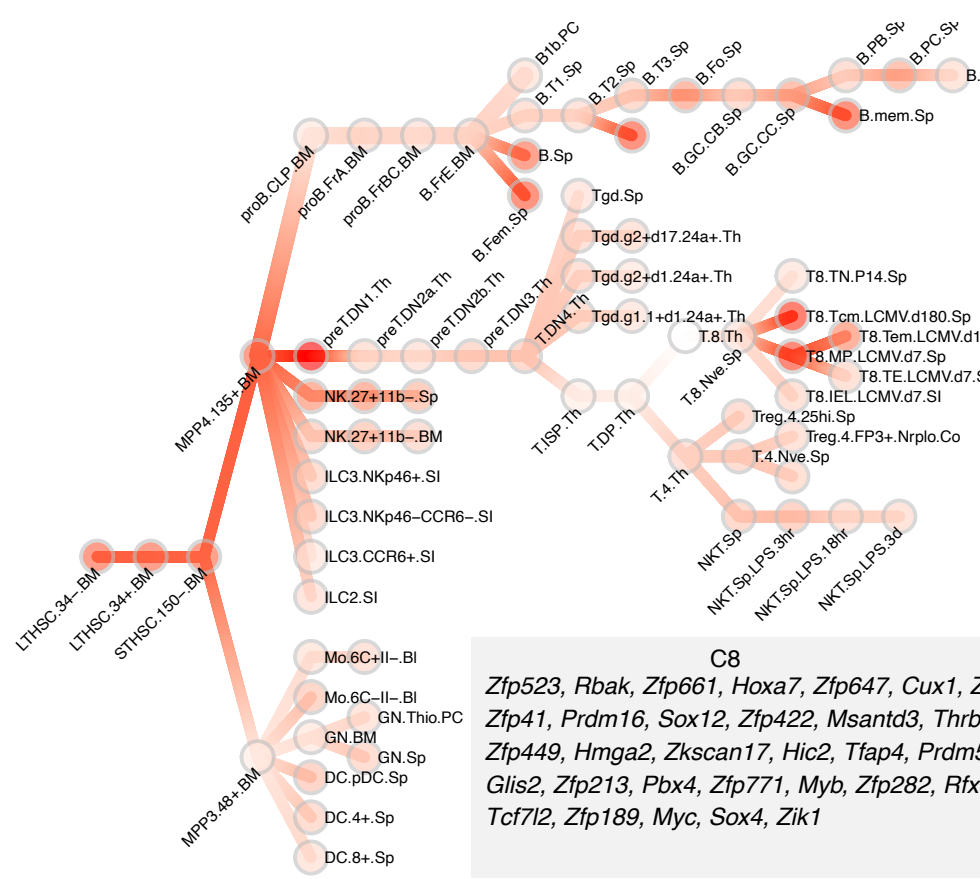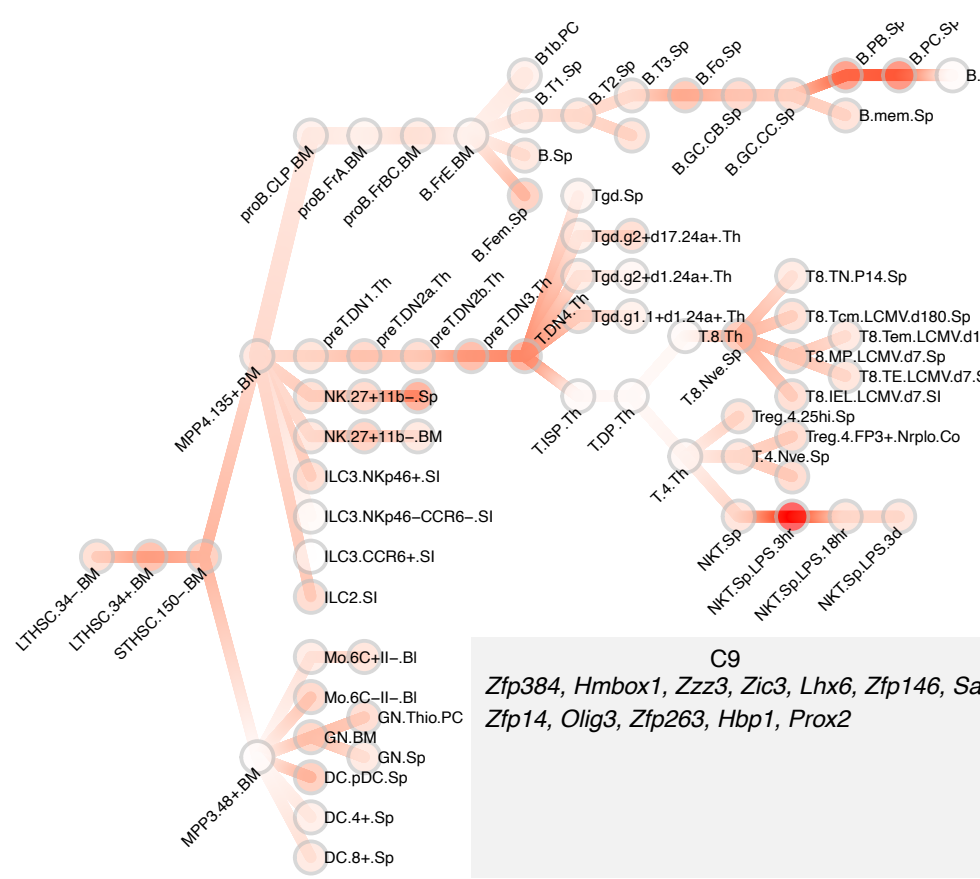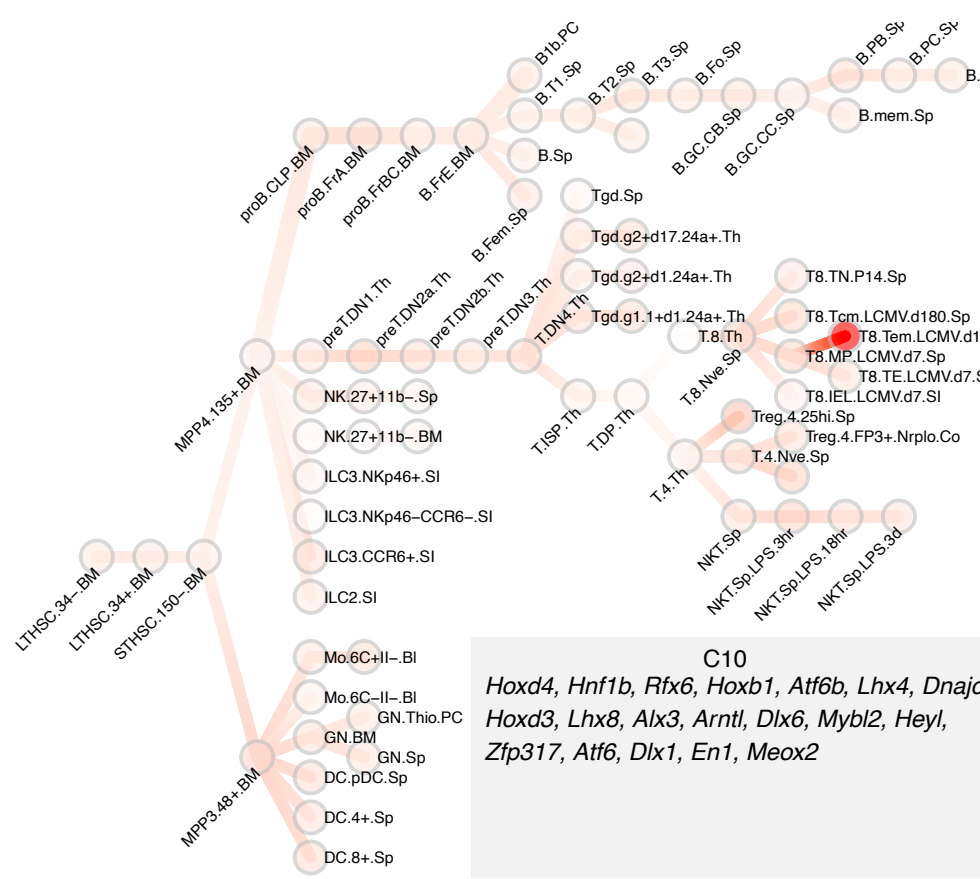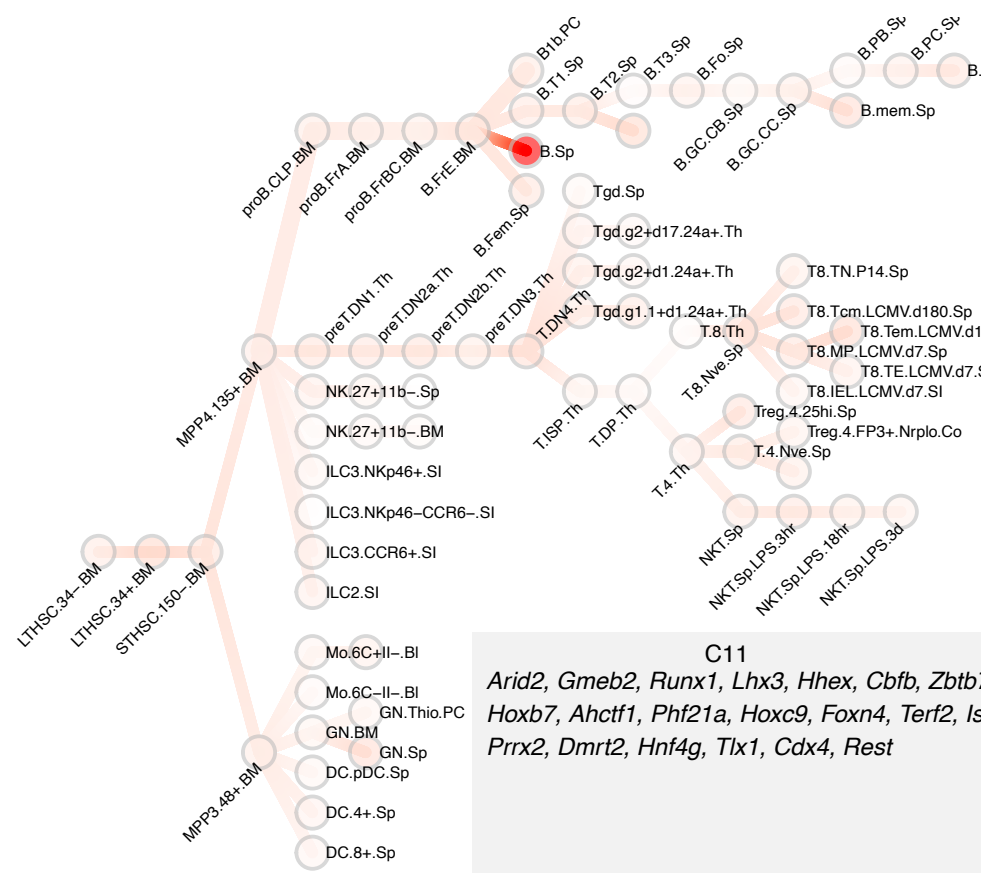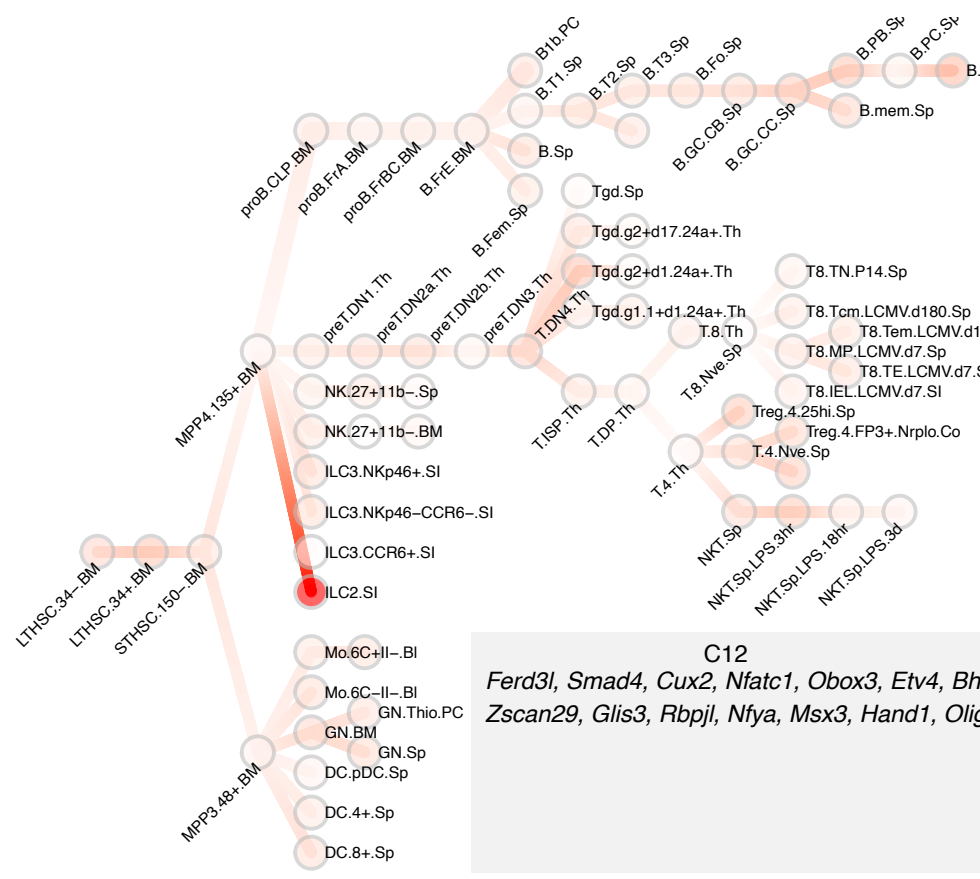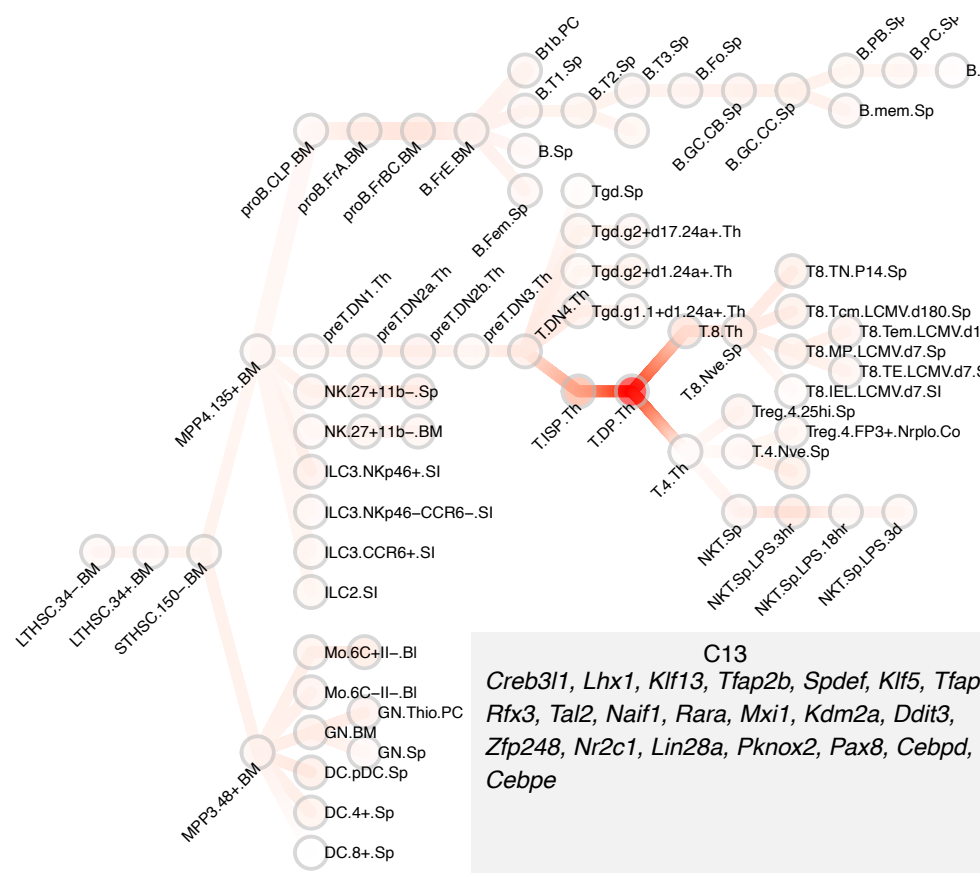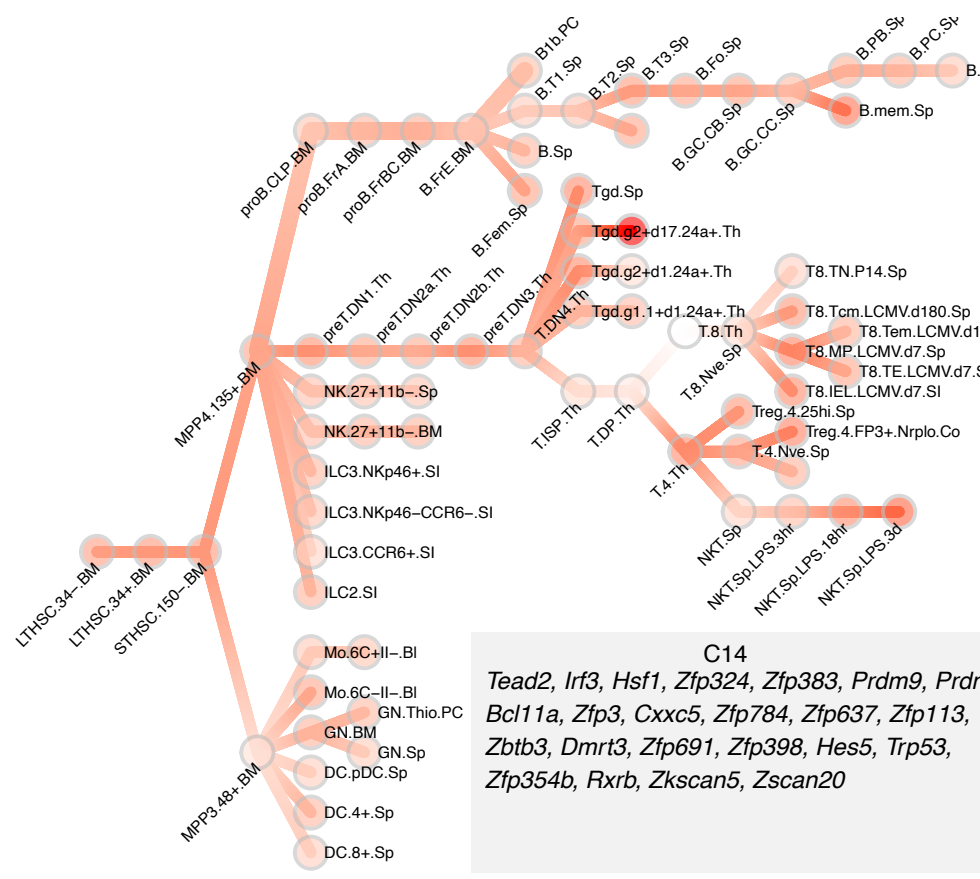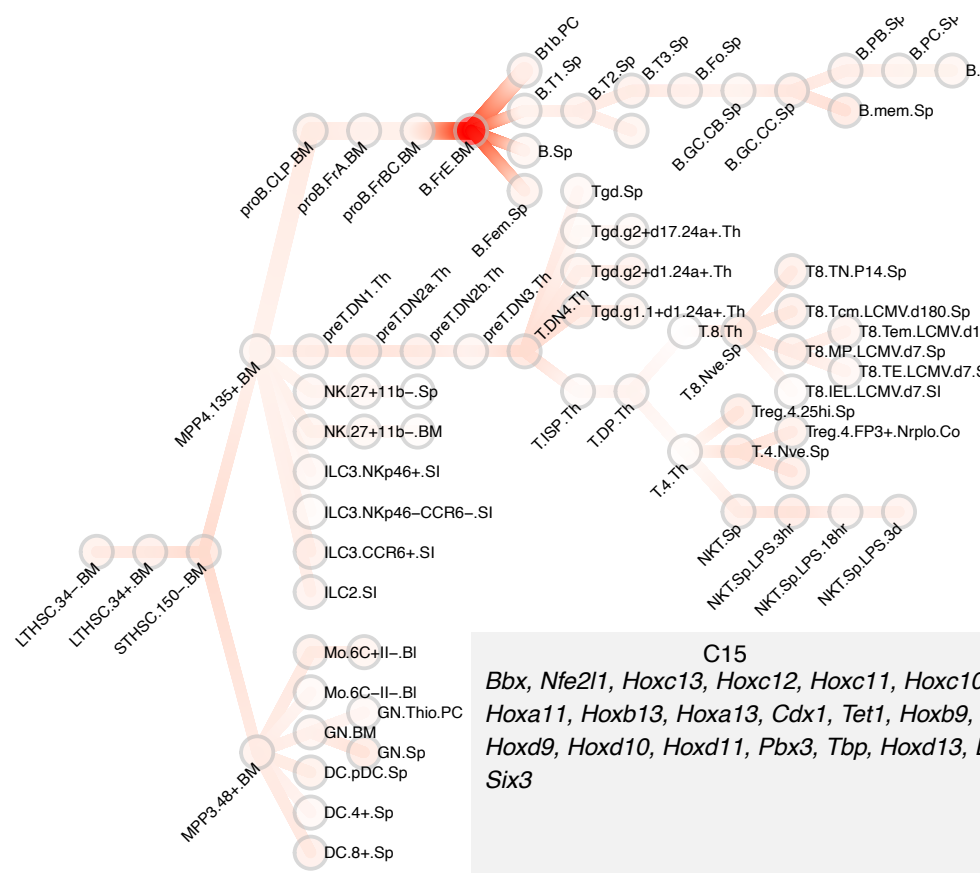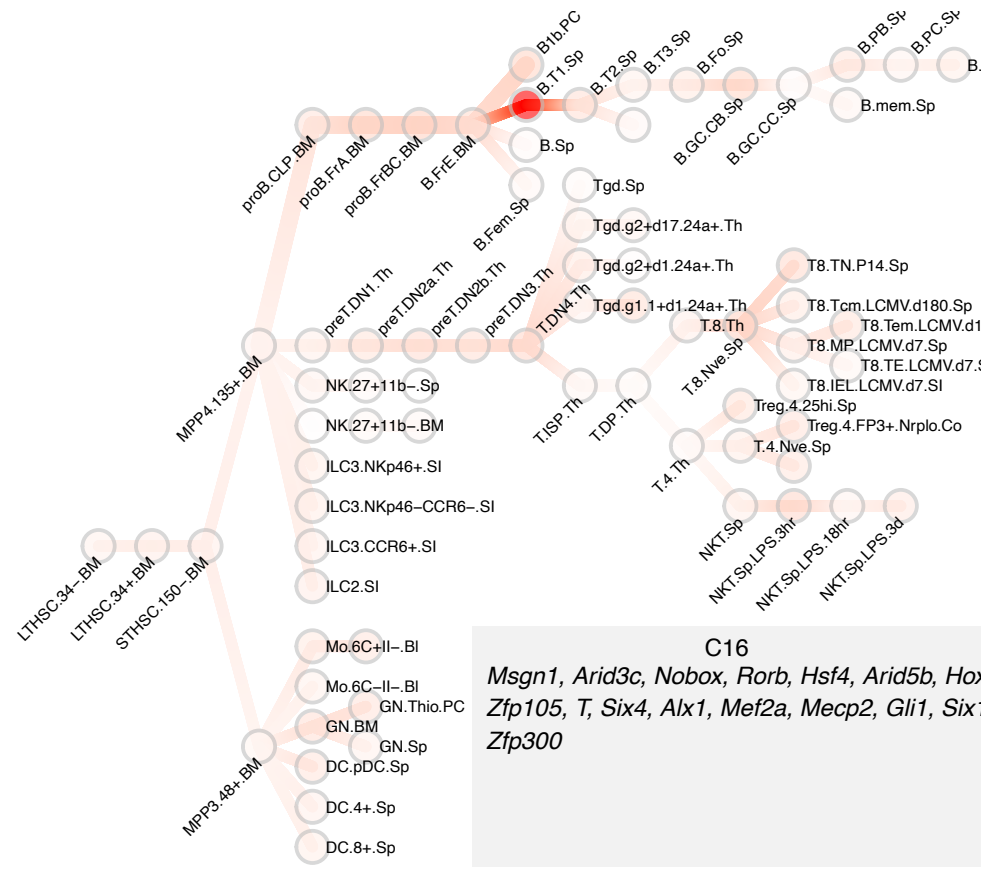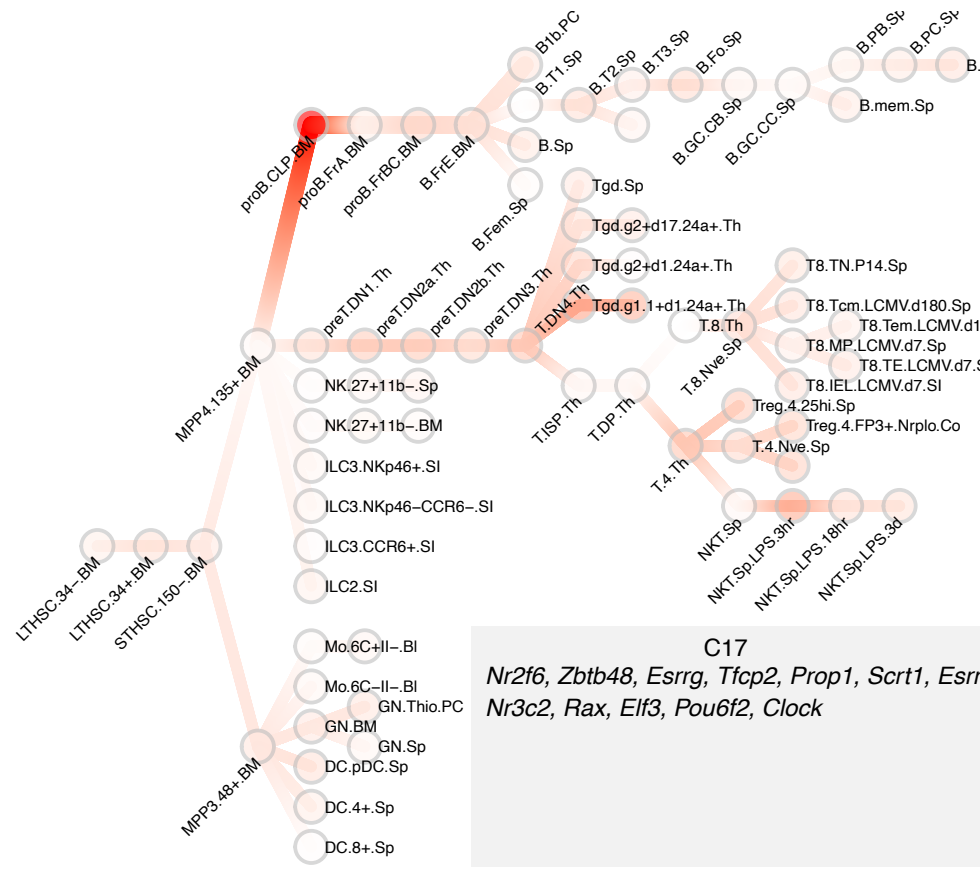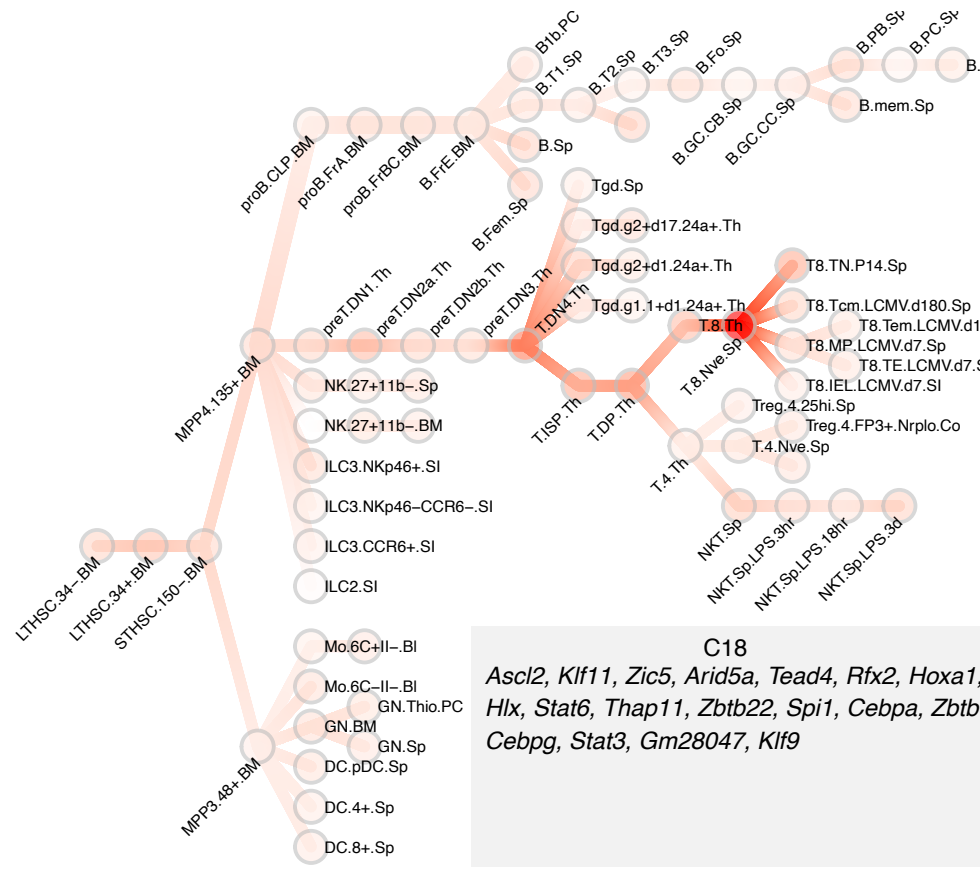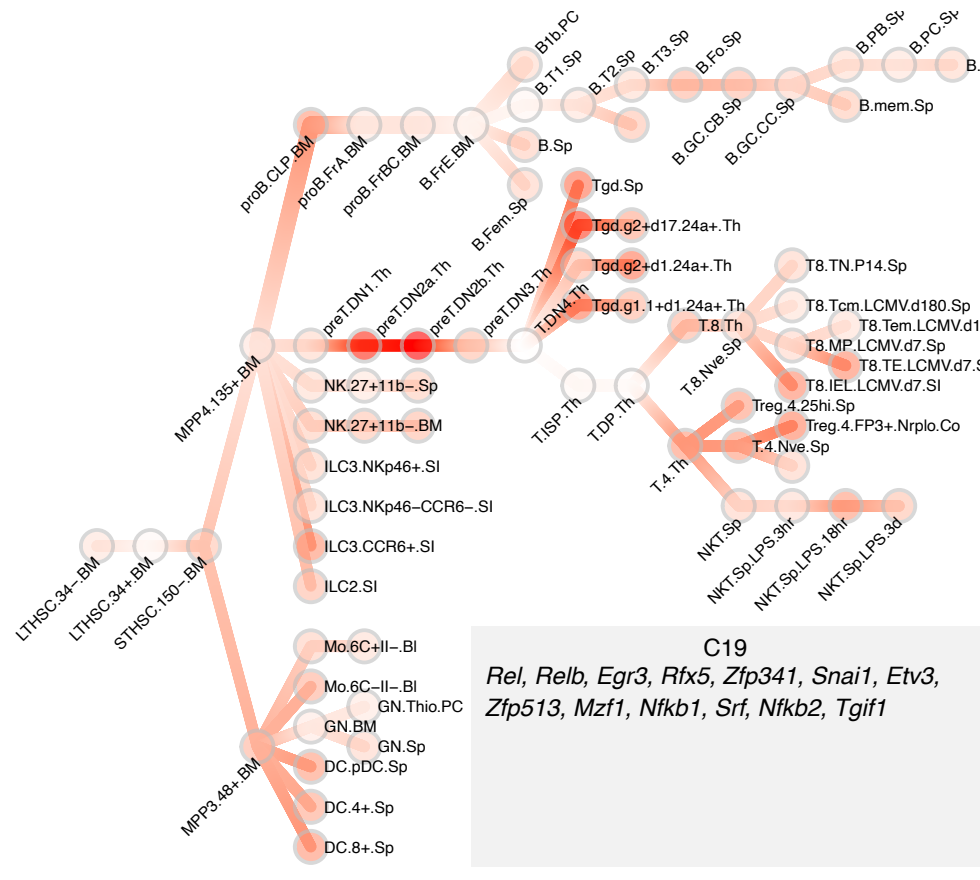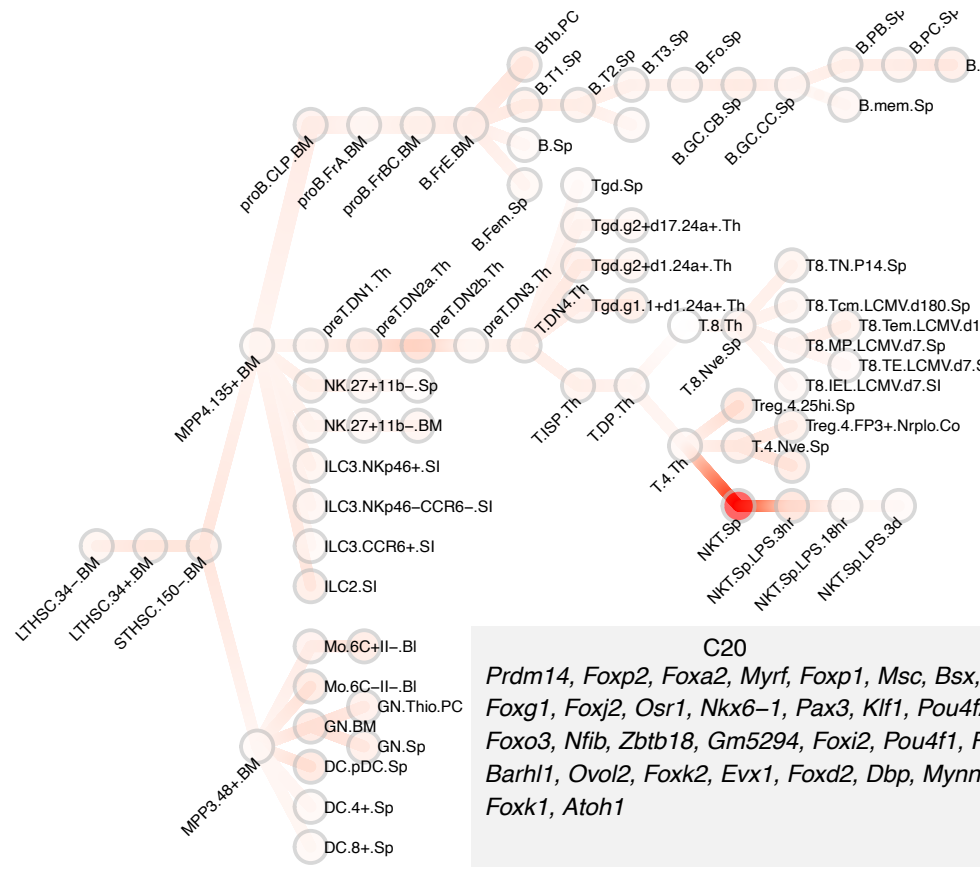

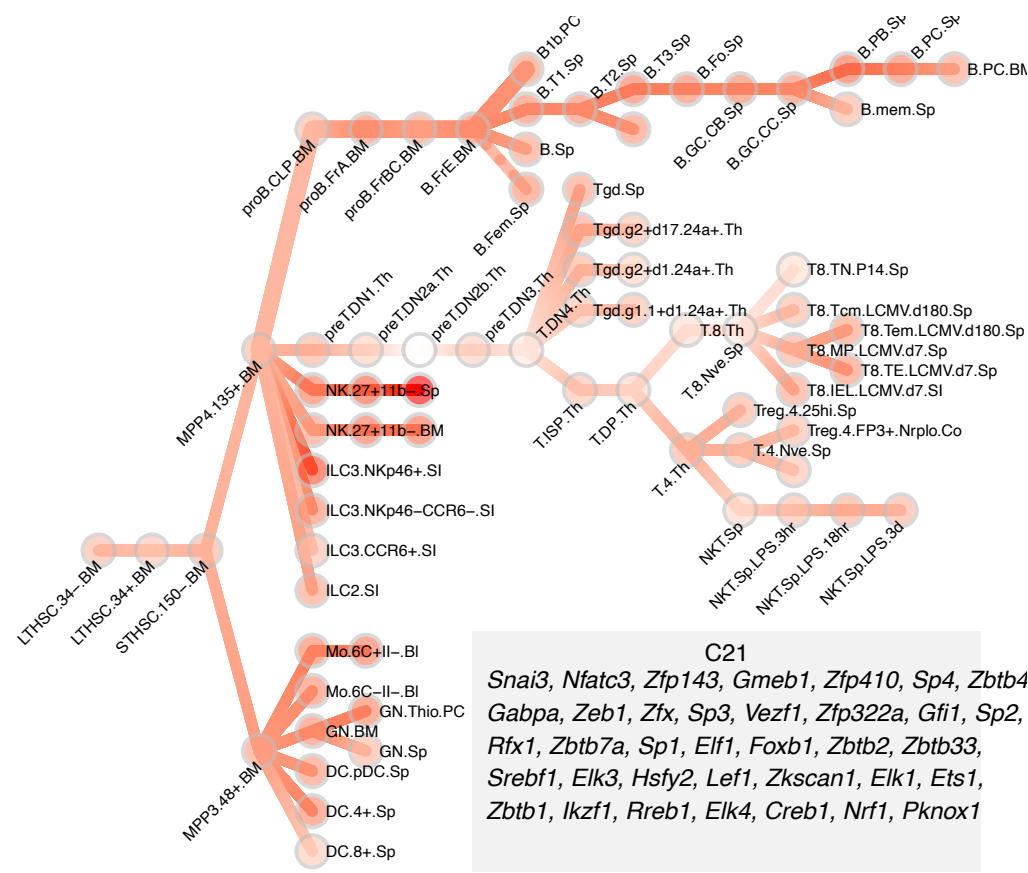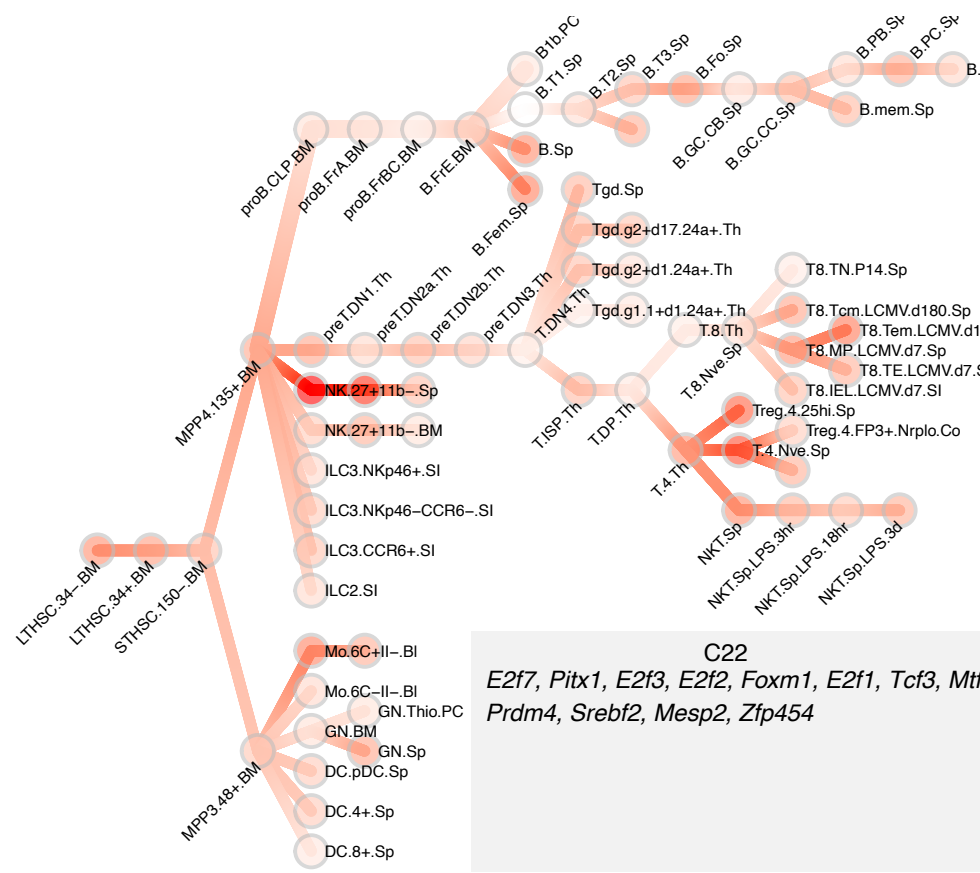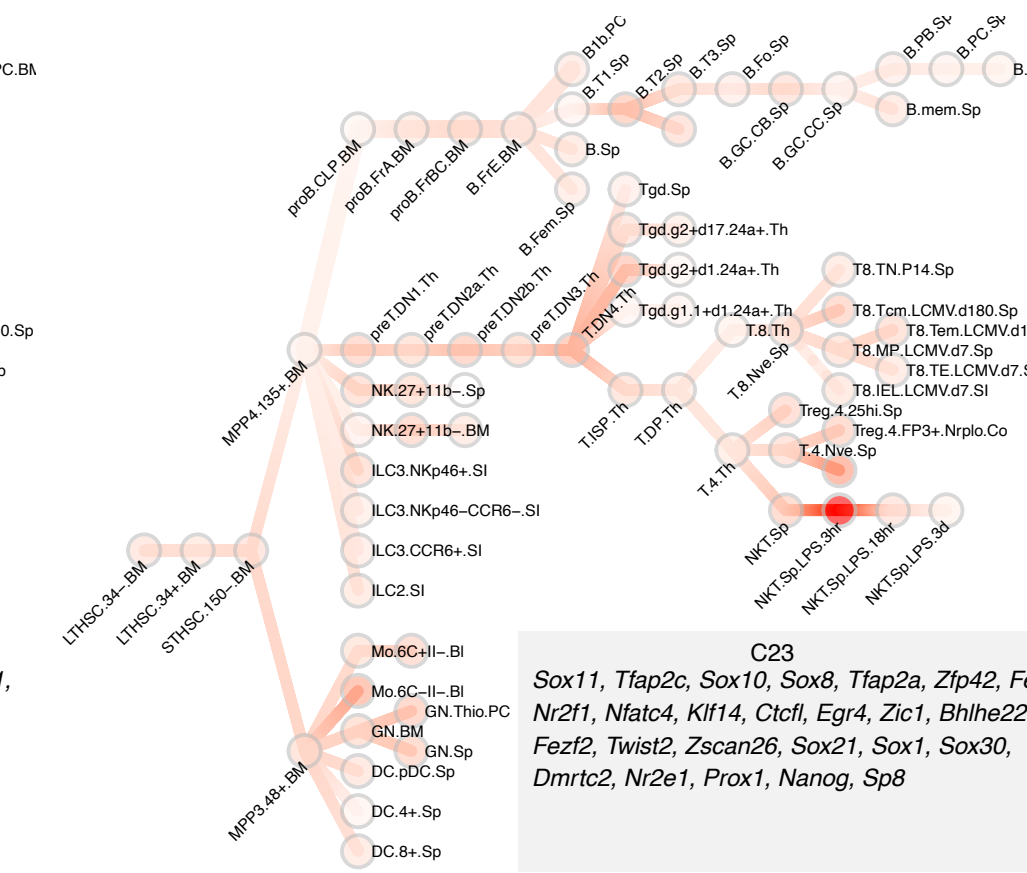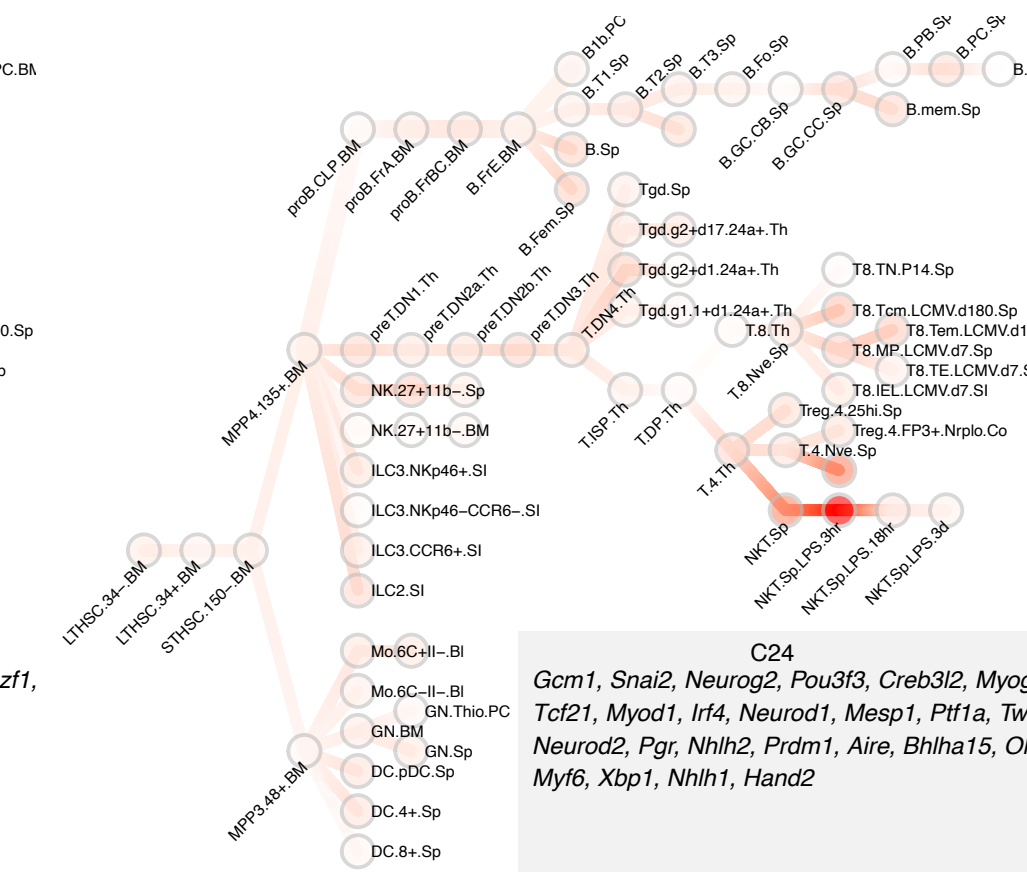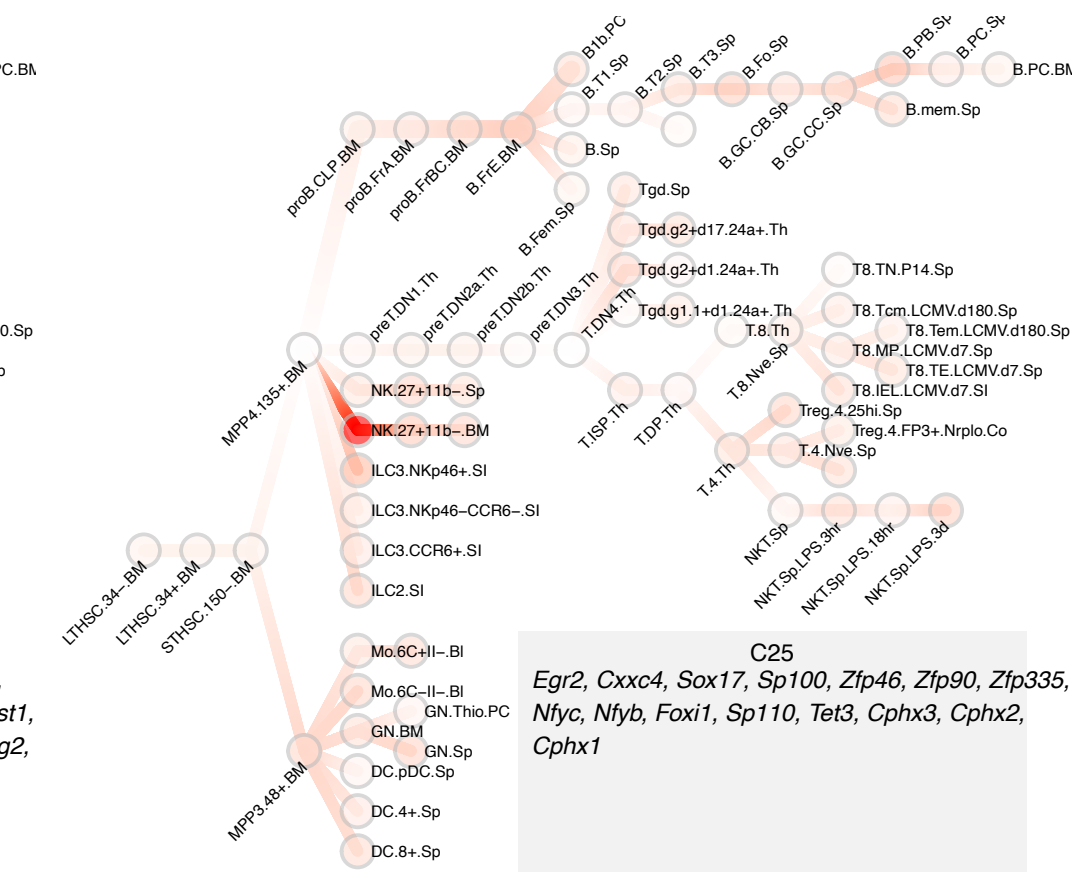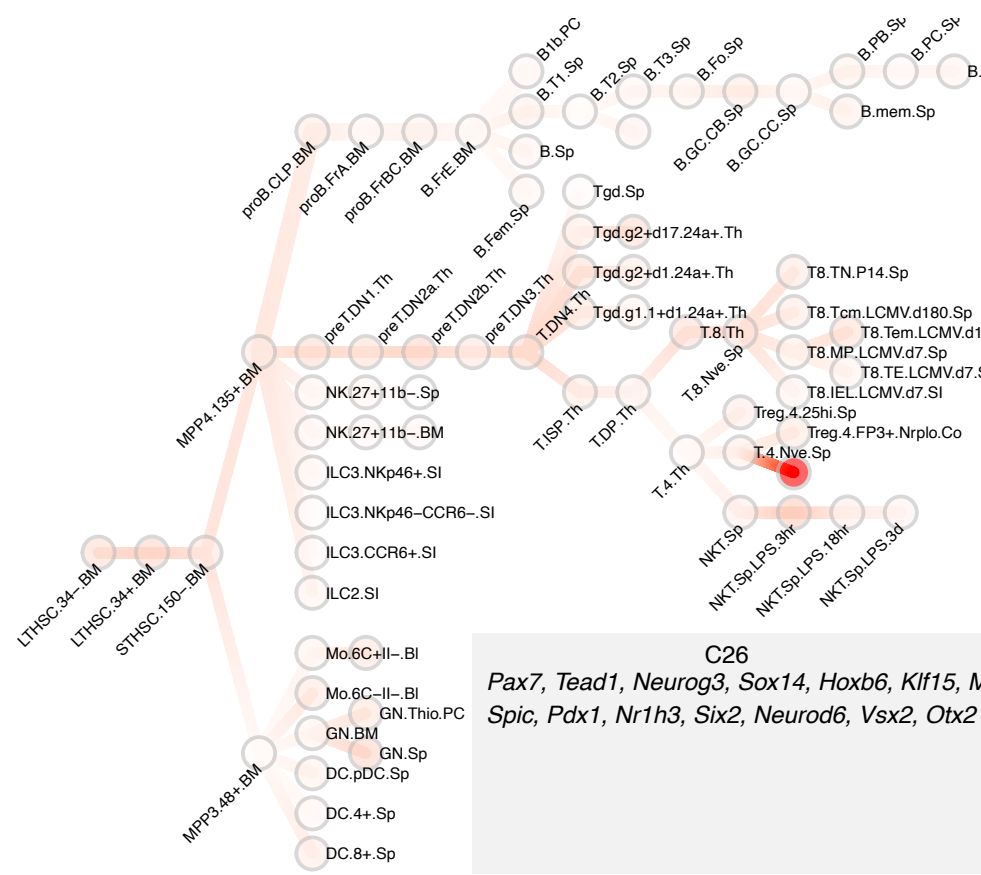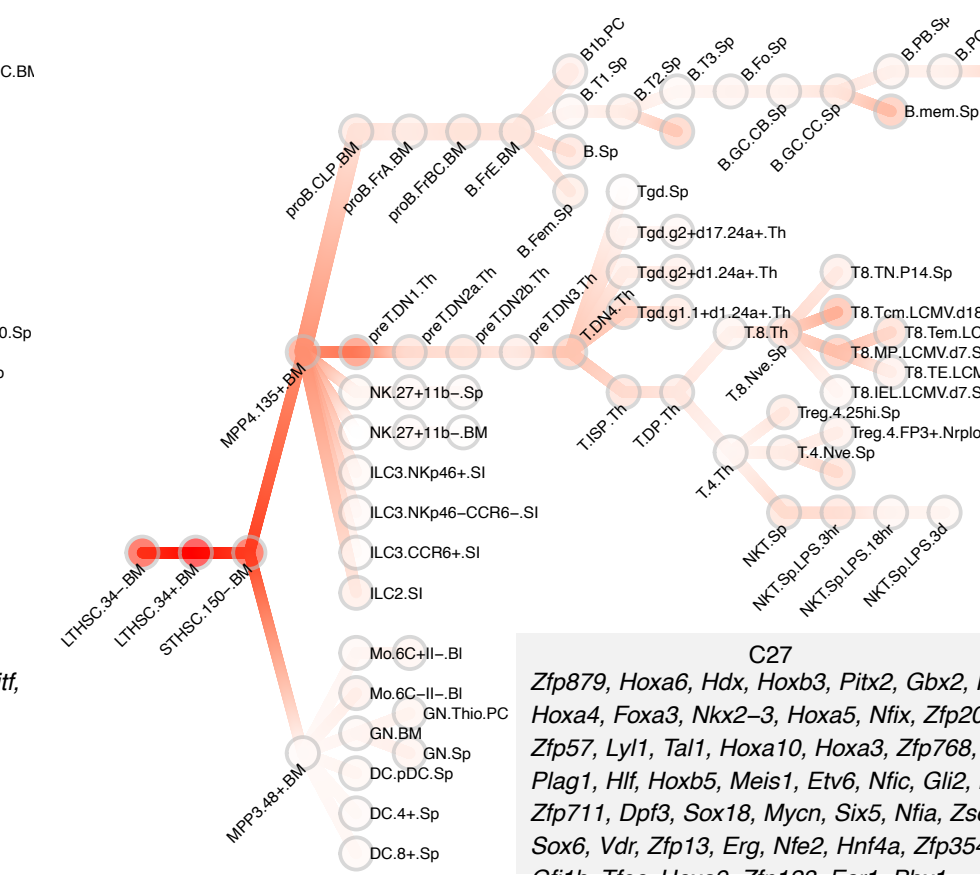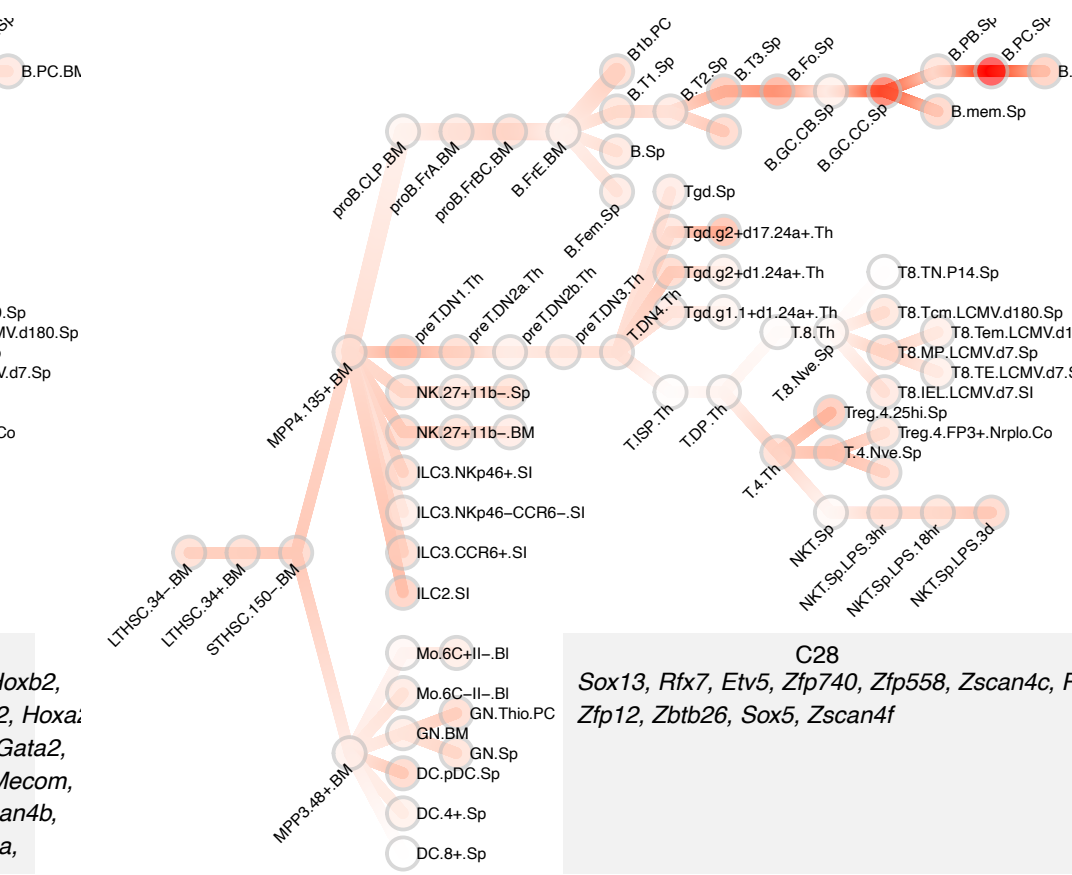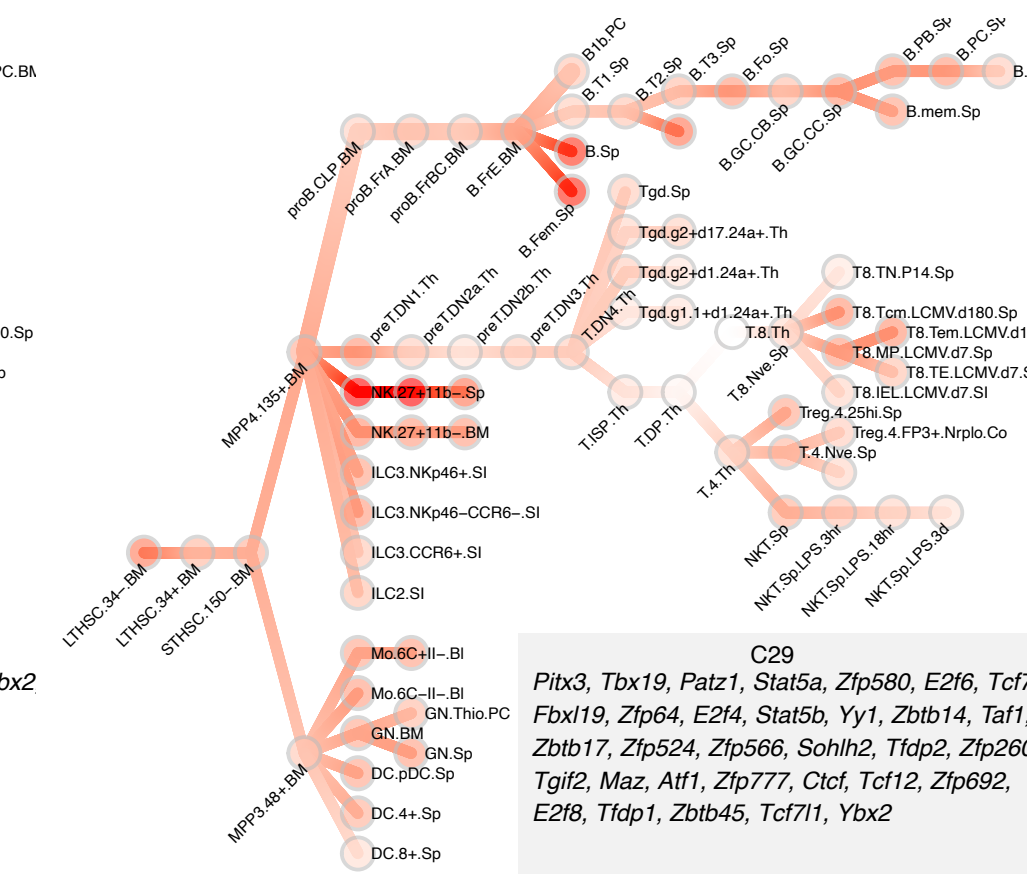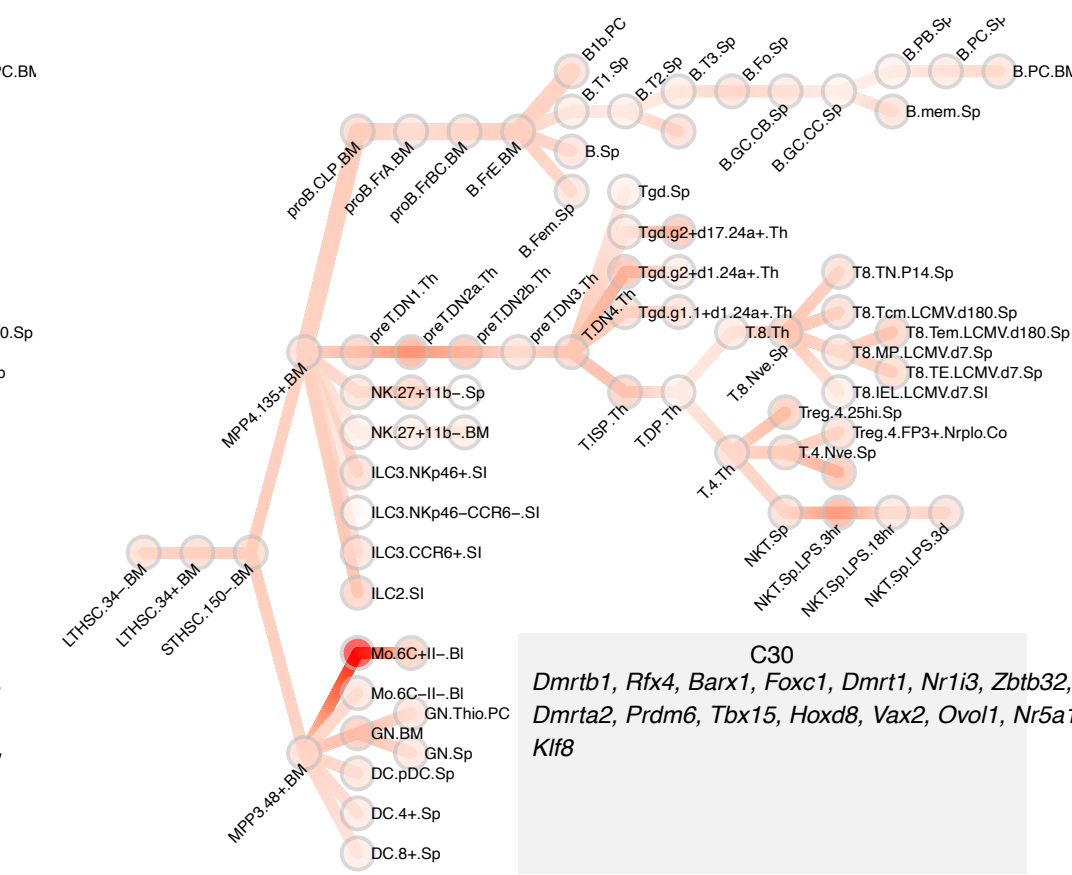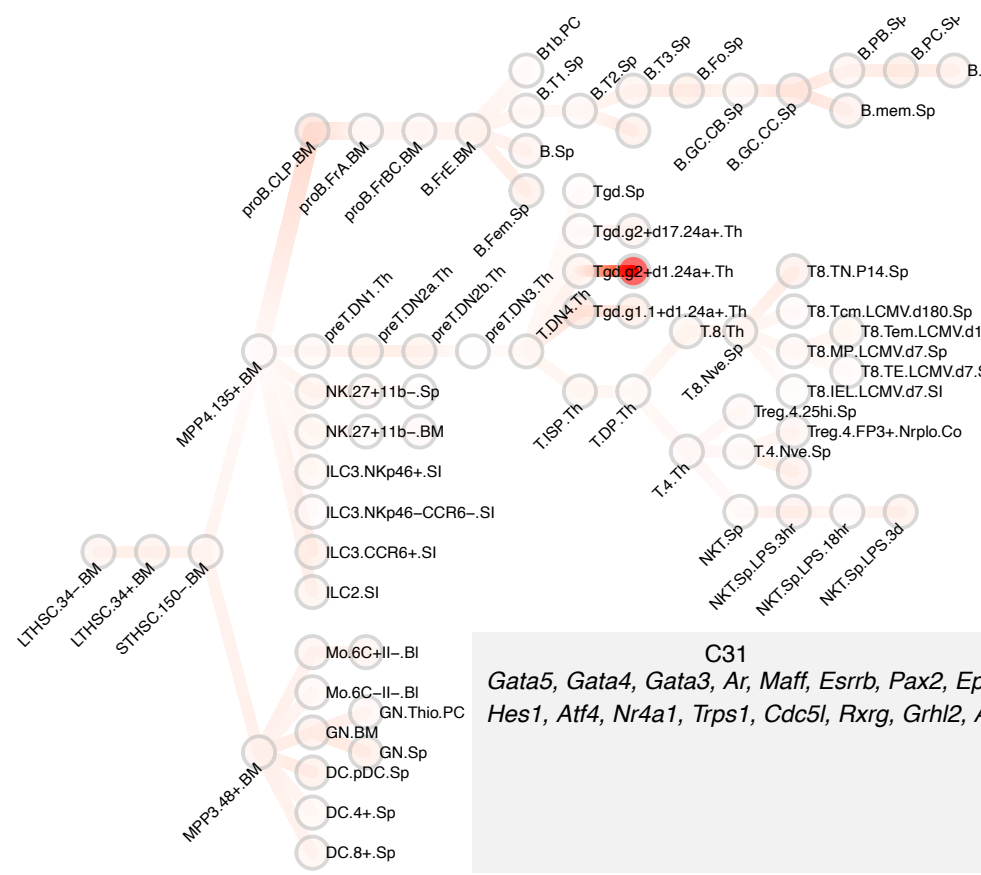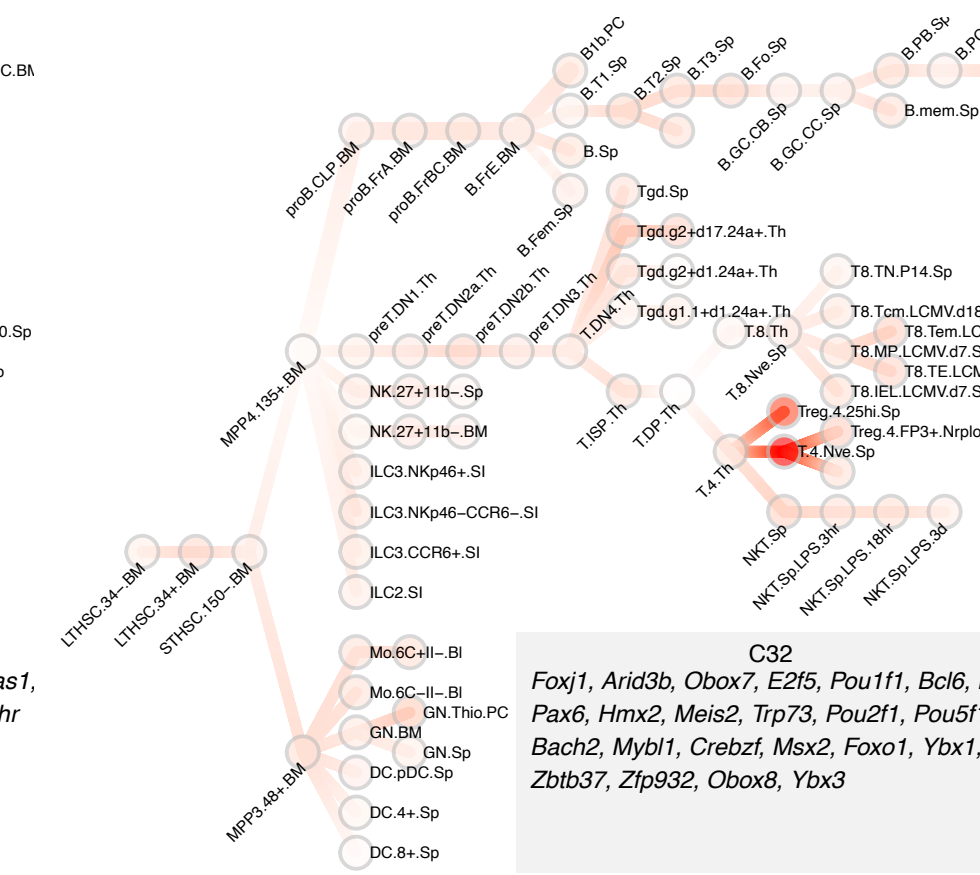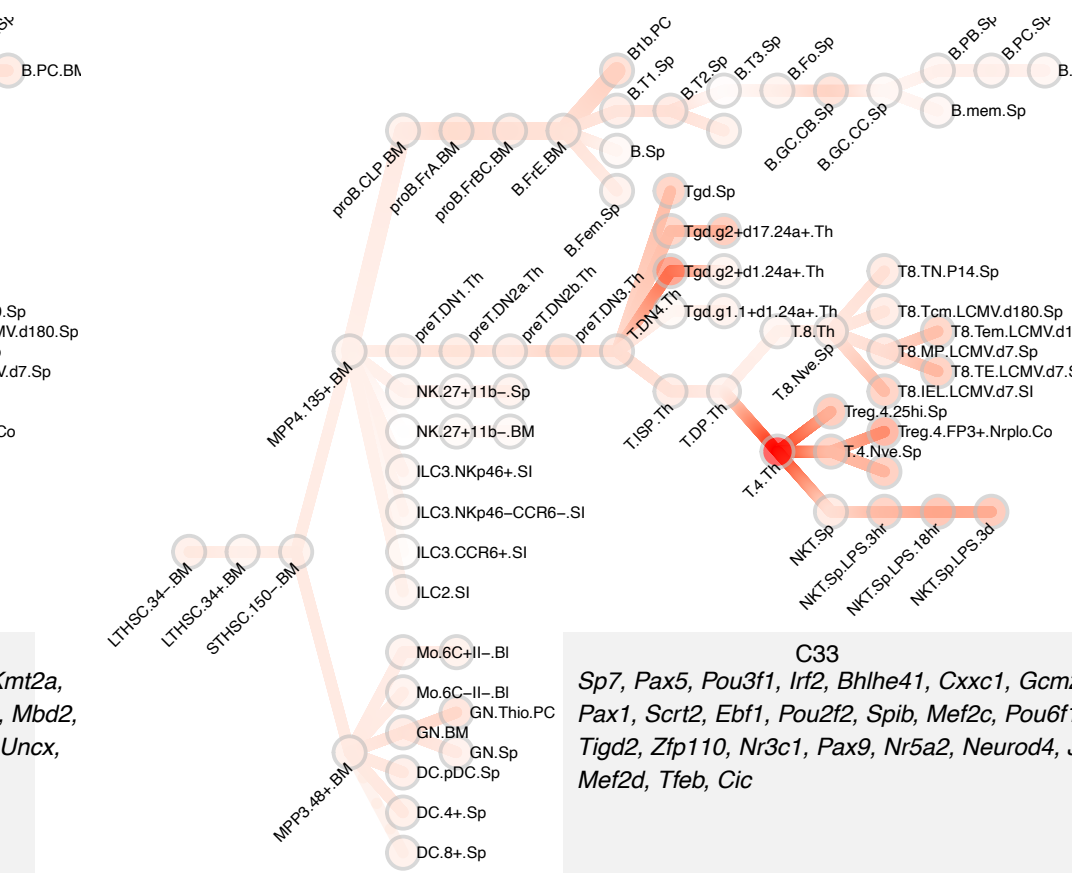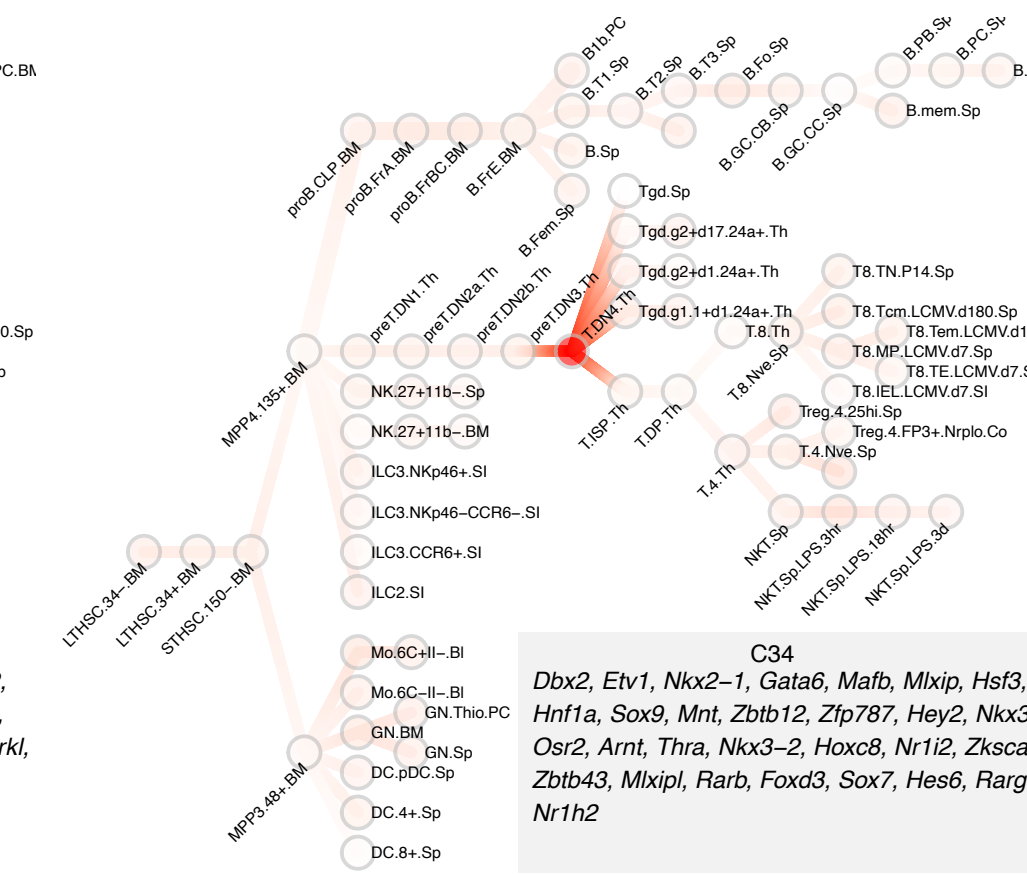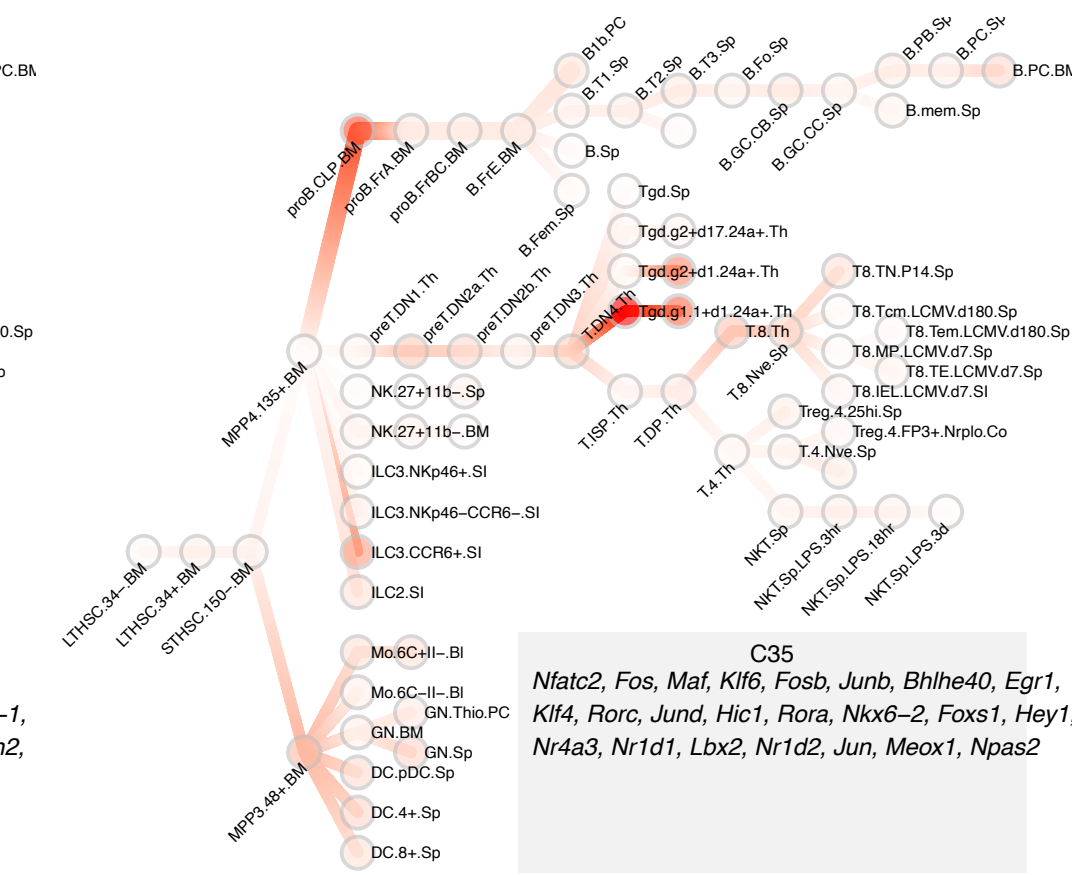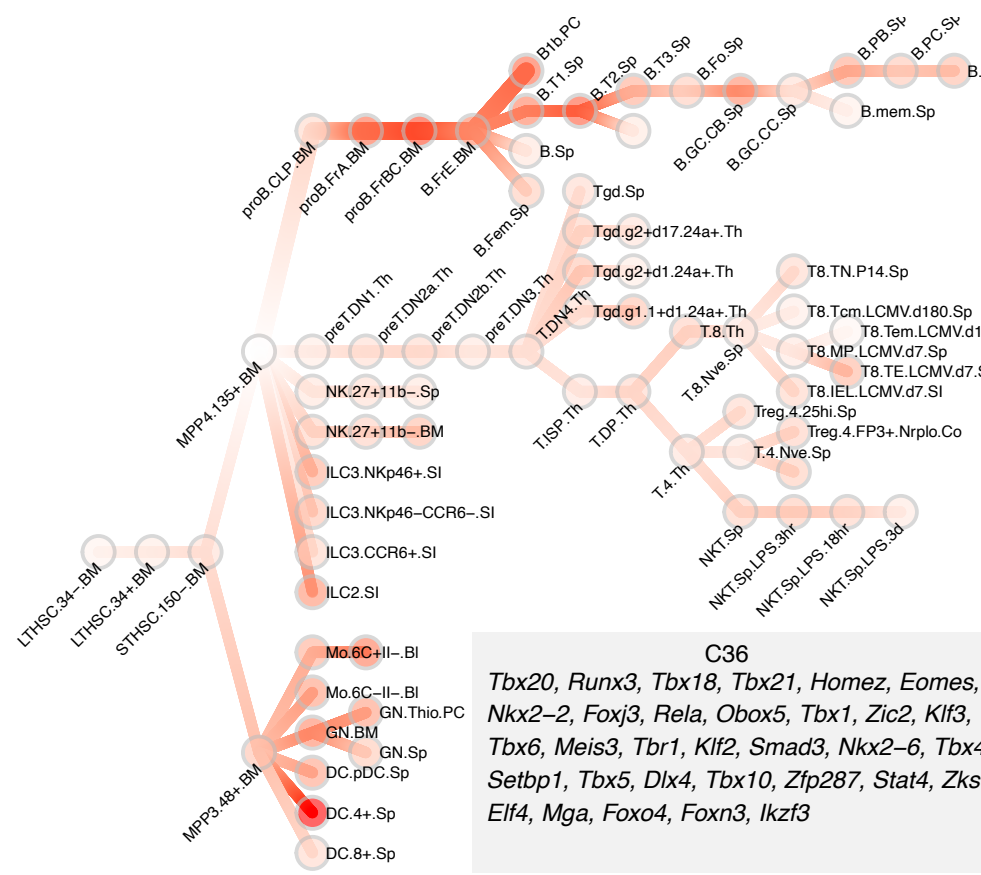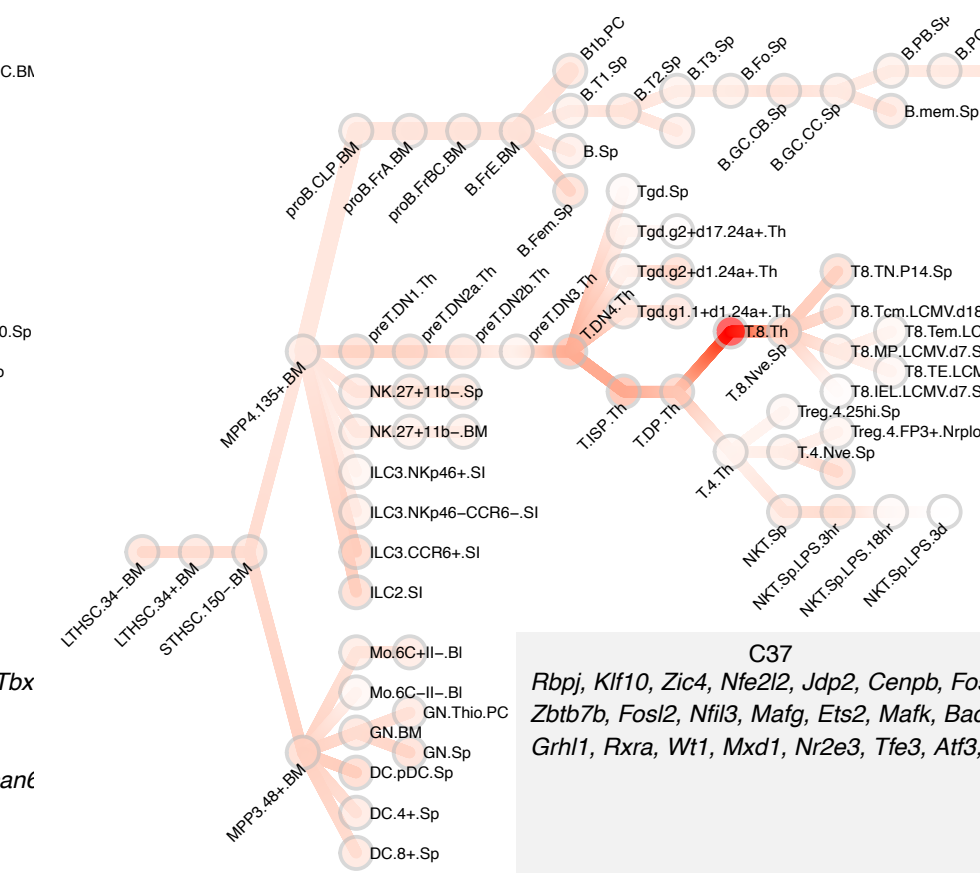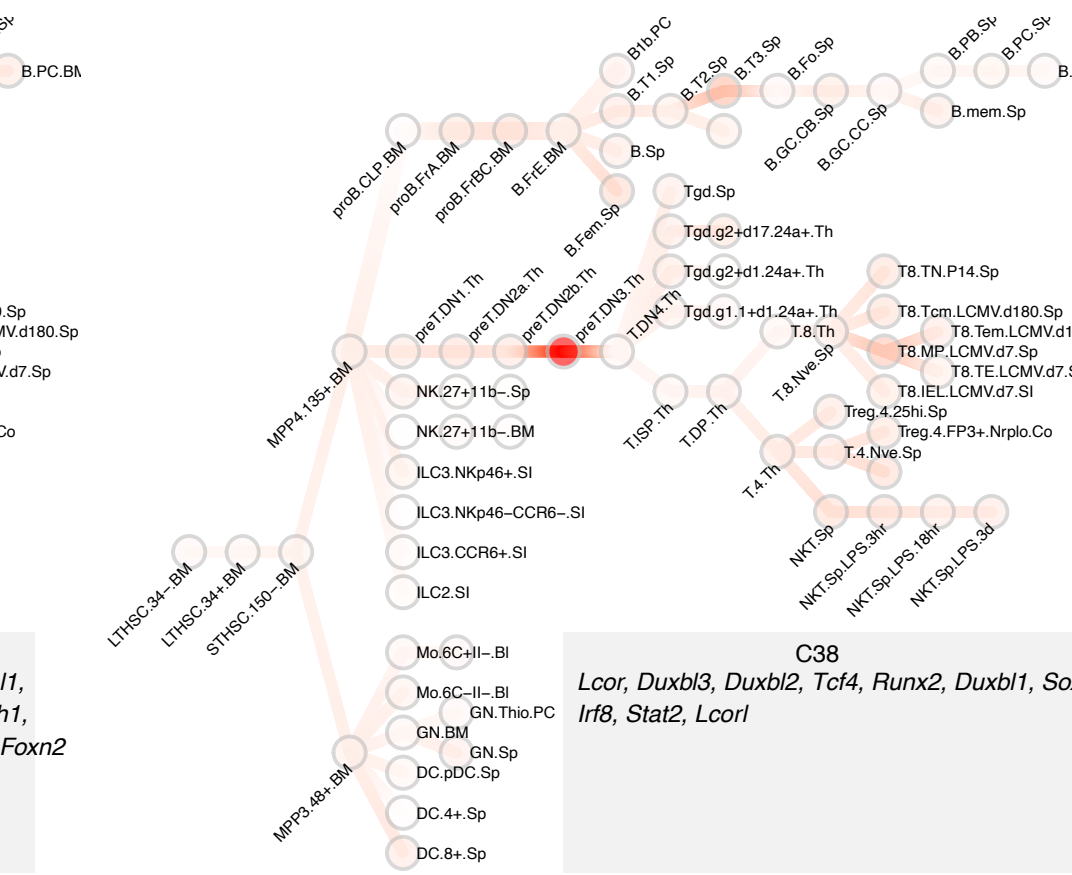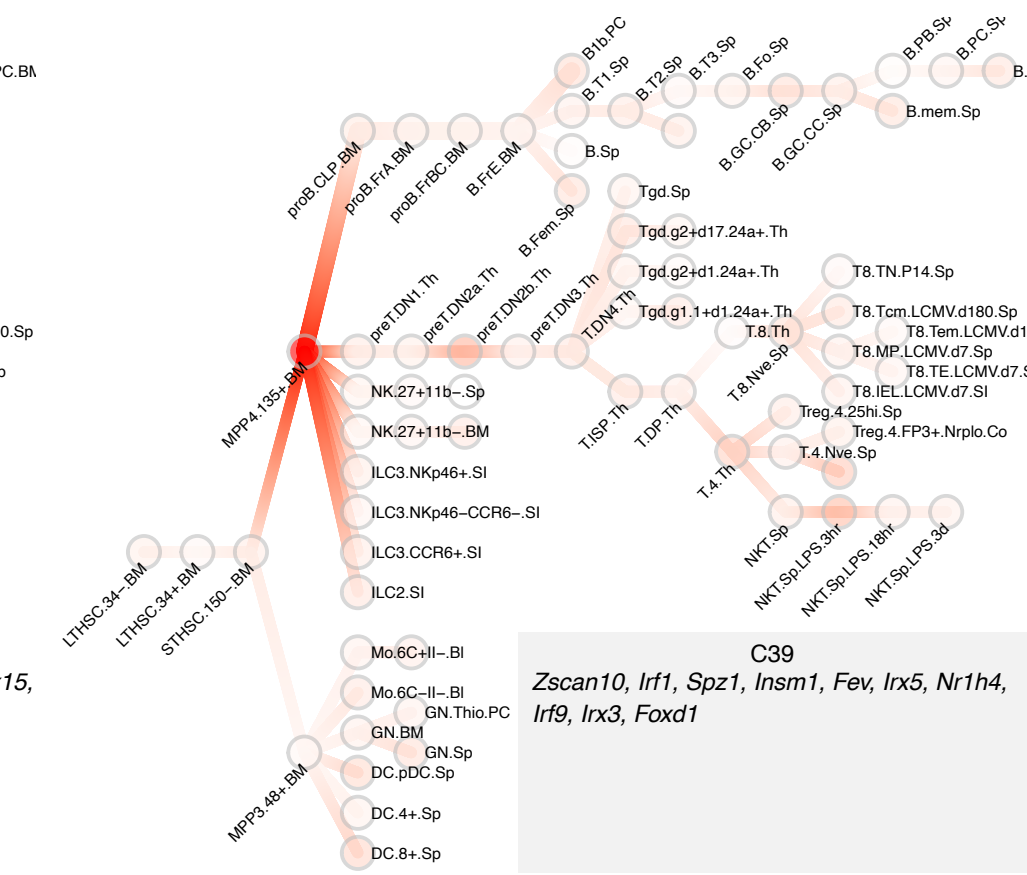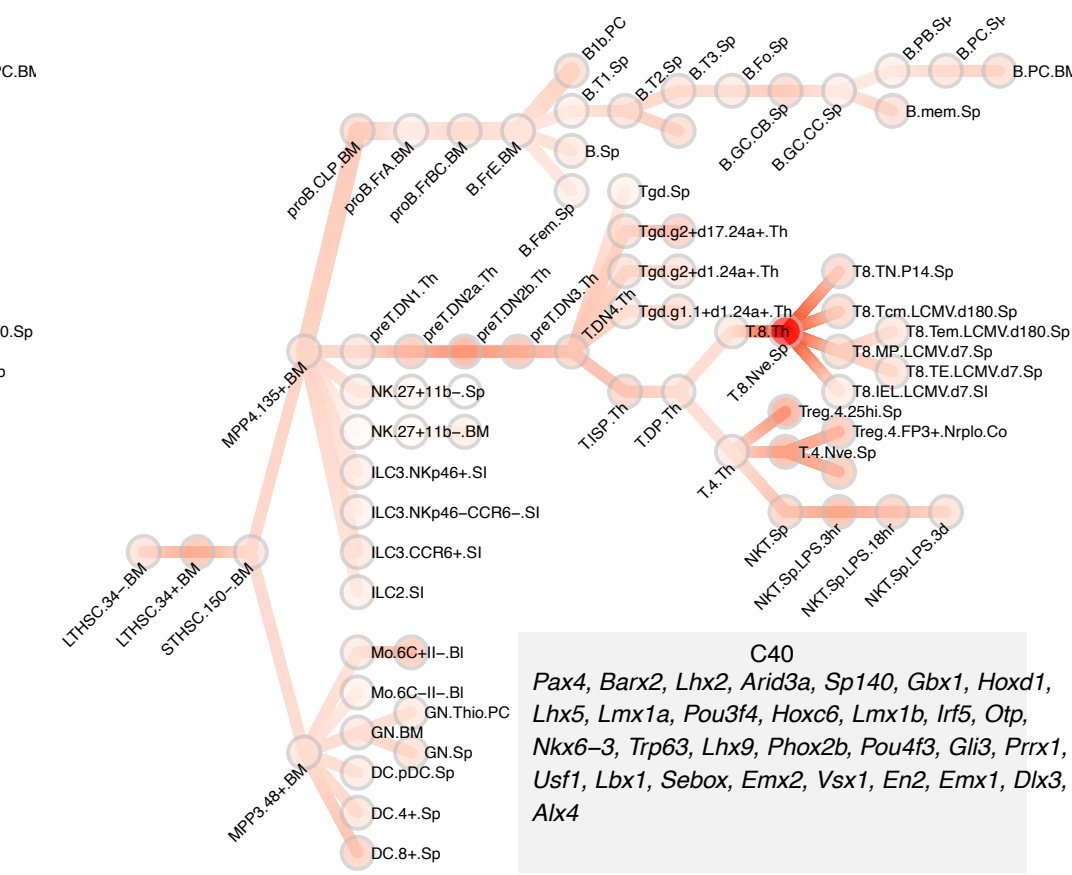

Supplement: S6 Fig — (PDF) [file pcbi.1010116.s006.pdf]

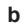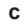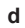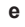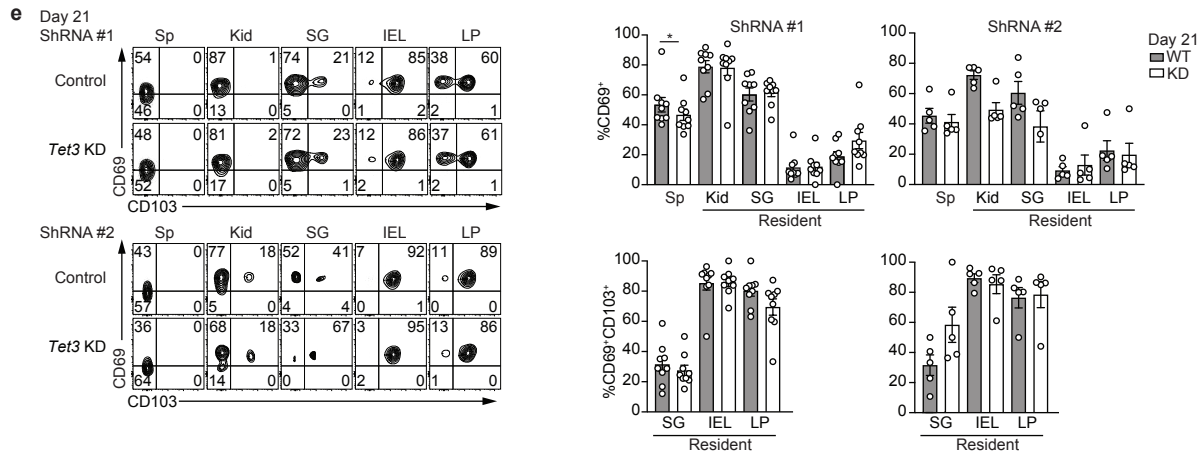

Supplement: S7 Fig — (A) qPCR analysis to assess knockdown efficiency of indicated gene in P14 cells prior to transfer. (B,C) Representative expression of CD69 and CD103 on Kdm2b KD and control P14 cells recovered from indicated tissue on day 7 and 21 of infection (left). Quantification of the frequency of populations (right). (D,E) Representative expression of CD69 and CD103 on Tet3 KD and control P14 cells recovered from indicated tissue on day 7 and 21 of infection (left). Quantification of the frequency of populations (right). Numbers in graphs indicate percent of cells in the corresponding gate. Data are cumulative of 2 (B,C) or 3 (D,E) independent experiments with n = 3–4. Graphs show mean ± SEM. A two-tailed ratio paired t-test was used to determine statistical significance; *p< 0.05, **p < 0.01. (PDF) [file pcbi.1010116.s007.pdf]
