# Supplementary material for: Systems-level identification of key transcription factors in immune cell specification
Source: PLoS Comput Biol. 2022 Sep 26;18(9):e1010116. doi: 10.1371/journal.pcbi.1010116 (PMC9536753; doi:10.1371/journal.pcbi.1010116)
Supplement: S4 Table — (DOCX) [file pcbi.1010116.s011.docx]

#### **Table S4. Predicted putative driver TFs in the five cell lineages**

B cell

| Predicted TFs | p-value | log2 fold change | Evidence |
| --- | --- | --- | --- |
| *Mef2c* | *1.66E-18* | *3.74* | Essential for B cell early development[[1]](https://paperpile.com/c/dsazkK/6O4W) |
| *Bcl11a* | *1.15E-15* | *3.55* | Relates to B cell development[[2]](https://paperpile.com/c/dsazkK/QqQfy) |
| *Tcf4* | *2.13E-15* | *2.01* | Essential for B cell early development[[3]](https://paperpile.com/c/dsazkK/S9OID) |
| *Irf8* | *2.65E-15* | *2.29* | Regulates to B cell differentiation[[4]](https://paperpile.com/c/dsazkK/FLKSa) |
| *Lyl1* | *2.79E-15* | *3.18* | Induces B-cell lymphoma[[5]](https://paperpile.com/c/dsazkK/ThKYB) |
| *Ebf1* | *1.07E-14* | *5.16* | Essential for B cell early development[[6]](https://paperpile.com/c/dsazkK/YL4XF) |
| *Pou2f2* | *5.30E-13* | *2.68* | Regulates B cell differentiation[[7]](https://paperpile.com/c/dsazkK/g9YtE) |
| *Spi1* | *4.82E-11* | *3.02* | Essential for B cell differentiation[[8]](https://paperpile.com/c/dsazkK/9nWDf) |
| *Zfp296* | *1.33E-10* | *1.67* | Unknown |
| *Pax5* | *1.44E-10* | *4.51* | Essential for B cell early development[[9]](https://paperpile.com/c/dsazkK/g3eY1) |

αβT cell

| Predicted TFs | p-value | log2 fold change | Evidence |
| --- | --- | --- | --- |
| *Tcf7* | *3.39E-11* | *3.48* | Relates to αβT cell differentiation[[10]](https://paperpile.com/c/dsazkK/j9c0A) |
| *Bcl11b* | *5.18E-10* | *2.84* | Required for αβT cell differentiation and survival[[11]](https://paperpile.com/c/dsazkK/KGzv6) |
| *Gata3* | *1.02E-7* | *2.11* | Essential for CD4^+^ T cell differentiation[[12]](https://paperpile.com/c/dsazkK/YHyxG) |
| *Tgif2* | *3.50E-6* | *0.68* | TGF-β–signaling components[[11]](https://paperpile.com/c/dsazkK/KGzv6) |
| *Zbtb1* | *3.64E-6* | *0.70* | Essential for development of T cells[[13,14]](https://paperpile.com/c/dsazkK/3Rs4Q+ZagIM) |
| *Lef1* | *7.27E-6* | *2.28* | Relates to αβT cell differentiation[[15]](https://paperpile.com/c/dsazkK/0kive) |
| *Zfp566* | *3.12E-5* | *0.63* | Unknown |
| *Stat5b* | *6.45E-5* | *0.65* | Promotes the expansion of mature αβT cells[[16,17]](https://paperpile.com/c/dsazkK/YwDPc+MvQ8k) |
| *Zfp64* | *2.34E-3* | *0.69* | Promotes Toll-like receptor-triggered innate immune response[[18,19]](https://paperpile.com/c/dsazkK/N7rpO+tj2FL) |
| *Stat5a* | *5.64E-3* | *0.78* | Indispensable in Treg cell development[[17,20]](https://paperpile.com/c/dsazkK/jhkPw+MvQ8k) |

Act T cell

| Predicted TFs | p-value | log2 fold change | Evidence |
| --- | --- | --- | --- |
| *Stat4* | *4.06E-9* | *1.20* | Required in CD4^+^ T helper cell differentiation[[21]](https://paperpile.com/c/dsazkK/hHYFZ) |
| *Bcl11b* | *4.29E-8* | *2.10* | Controls CD8^+^ T cell fate decisions[[22]](https://paperpile.com/c/dsazkK/tddix) |
| *Tbx21* | *1.97E-7* | *3.40* | Orchestrate Th1, Th2, and CD8^+^ T cell differentiation[[23,24]](https://paperpile.com/c/dsazkK/H4dMZ+GiJQO) |
| *Gata3* | *3.24E-6* | *1.74* | Controls Th2, CD8^+^ T cell proliferation[[25]](https://paperpile.com/c/dsazkK/MAiJD) |
| *Snai3* | *1.43E-5* | *1.93* | Regulate CD8^+^ T cell activation[[26]](https://paperpile.com/c/dsazkK/g7qJf) |
| *Lef1* | *2.68E-5* | *1.66* | Regulate CD8^+^ T cell differentiation[[27]](https://paperpile.com/c/dsazkK/o6T8D) |
| *Ikzf3* | *3.89E-5* | *1.30* | Controls CD8^+^ T cell responses[[28]](https://paperpile.com/c/dsazkK/l3Aiv) |
| *Hic1* | *1.16E-4* | *1.36* | Critical in T cell function regulation[[29]](https://paperpile.com/c/dsazkK/N7Zfq) |
| *Batf* | *3.04E-4* | *1.50* | Essential checkpoint in early effector CD8^+^ T cell[[30]](https://paperpile.com/c/dsazkK/yp5GZ) |
| *Eomes* | *3.54E-4* | *2.43* | Relates to T cell activation[[31]](https://paperpile.com/c/dsazkK/MWRFb) |

γδT cell

| Predicted TFs | p-value | log2 fold change | Evidence |
| --- | --- | --- | --- |
| *Tcf7* | *1.22E-10* | *3.18* | γδT cell-specific gene[[32]](https://paperpile.com/c/dsazkK/igFzp) |
| *Ar* | *1.27E-8* | *1.78* | Unknown |
| *Snai3* | *4.56E-7* | *1.99* | Unknown |
| *Bcl11b* | *9.22E-7* | *2.31* | Relates to γδT cell development[[33]](https://paperpile.com/c/dsazkK/zMC0A) |
| *Gata3* | *1.19E-5* | *2.35* | Correlates with γδT cell functions[[34]](https://paperpile.com/c/dsazkK/7r6I9) |
| *Sox13* | *2.56E-5* | *3.48* | γδT cell-specific gene[[32]](https://paperpile.com/c/dsazkK/igFzp) |
| *Ikzf3* | *6.77E-5* | *1.12* | Correlates with γδT cell functions[[35]](https://paperpile.com/c/dsazkK/yHr8y) |
| *Lef1* | *1.09E-4* | *2.27* | γδT cell-specific gene[[32]](https://paperpile.com/c/dsazkK/igFzp) |
| *Egr2* | *1.32E-4* | *1.28* | Relates to γδT cell expansion[[36,37]](https://paperpile.com/c/dsazkK/BhkxJ+sbrzj) |
| *Rorc* | *6.61E-4* | *2.74* | Required for γδT cell specification and differentiation[[38]](https://paperpile.com/c/dsazkK/YeSKV) |

ILC cell

| Predicted TFs | p-value | log2 fold change | Evidence |
| --- | --- | --- | --- |
| *Fosl2* | *4.48E-11* | *1.93* | Necessary for NK cell differentiation[[39]](https://paperpile.com/c/dsazkK/vXNYK) |
| *Runx*3 | *7.11E-10* | *1.30* | Regulates NK cell activation[[40]](https://paperpile.com/c/dsazkK/MWPJA) |
| *Meis*1 | *1.28E-7* | *1.42* | Unknown |
| *Pbx*3 | *5.37E-7* | *1.14* | Unknown |
| *Bhlhe*40 | *1.95E-6* | *1.93* | Required for NK cell normal functions[[41]](https://paperpile.com/c/dsazkK/tuY5f) |
| *Gata*3 | *3.25E-6* | *2.39* | Promotes NK cell differentiation[[42]](https://paperpile.com/c/dsazkK/CH2Li) |
| *Nfil*3 | *3.92E-6* | *1.41* | Essential for maintenance of NK cells[[43]](https://paperpile.com/c/dsazkK/i0v0P) |
| *Jdp*2 | *8.92E-6* | *1.04* | Unknown |
| *Zscan*17 | *1.62E-5* | *2.34* | Unknown |
| *Stat*4 | *1.67E-5* | *1.16* | Induces NK cell tolerance in lung diseases[[44]](https://paperpile.com/c/dsazkK/DPX7h) |

####

#### Reference

1. [Herglotz J, Unrau L, Hauschildt F, Fischer M, Kriebitzsch N, Alawi M, et al. Essential control of early B-cell development by Mef2 transcription factors. Blood. 2016;127: 572–581.](http://paperpile.com/b/dsazkK/6O4W)

2. [Lee B-S, Lee B-K, Iyer VR, Sleckman BP, Shaffer AL, III, et al. Corrected and Republished from: BCL11A Is a Critical Component of a Transcriptional Network That Activates Recombinase Activating Gene Expression and V(D)J Recombination. Mol Cell Biol. 2018;38. doi:](http://paperpile.com/b/dsazkK/QqQfy)[10.1128/MCB.00362-17](http://dx.doi.org/10.1128/MCB.00362-17)

3. [Wöhner M, Tagoh H, Bilic I, Jaritz M, Poliakova DK, Fischer M, et al. Molecular functions of the transcription factors E2A and E2-2 in controlling germinal center B cell and plasma cell development. J Exp Med. 2016;213: 1201–1221.](http://paperpile.com/b/dsazkK/S9OID)

4. [Vipul S, Runqing L. IRF4 and IRF8: Governing the virtues of B Lymphocytes. Front Biol. 2014;9: 269.](http://paperpile.com/b/dsazkK/FLKSa)

5. [Zhong Y, Jiang L, Hiai H, Toyokuni S, Yamada Y. Overexpression of a transcription factor LYL1 induces T- and B-cell lymphoma in mice. Oncogene. 2007;26: 6937–6947.](http://paperpile.com/b/dsazkK/ThKYB)

6. [Nechanitzky R, Akbas D, Scherer S, Györy I, Hoyler T, Ramamoorthy S, et al. Transcription factor EBF1 is essential for the maintenance of B cell identity and prevention of alternative fates in committed cells. Nat Immunol. 2013;14: 867–875.](http://paperpile.com/b/dsazkK/YL4XF)

7. [Hodson DJ, Shaffer AL, Xiao W, Wright GW, Schmitz R, Phelan JD, et al. Regulation of normal B-cell differentiation and malignant B-cell survival by OCT2. Proc Natl Acad Sci U S A. 2016;113: E2039.](http://paperpile.com/b/dsazkK/g9YtE)

8. [Ying H, Chang J-F, Parnes JR. PU.1/Spi-1 Is Essential for the B Cell-Specific Activity of the Mouse CD72 Promoter. The Journal of Immunology. 1998;160: 2287–2296.](http://paperpile.com/b/dsazkK/9nWDf)

9. [Cobaleda C, Schebesta A, Delogu A, Busslinger M. Pax5: the guardian of B cell identity and function. Nat Immunol. 2007;8: 463–470.](http://paperpile.com/b/dsazkK/g3eY1)

10. [Okamura RM, Sigvardsson M, Galceran J, Verbeek S, Clevers H, Grosschedl R. Redundant regulation of T cell differentiation and TCRalpha gene expression by the transcription factors LEF-1 and TCF-1. Immunity. 1998;8. doi:](http://paperpile.com/b/dsazkK/j9c0A)[10.1016/s1074-7613(00)80454-9](http://dx.doi.org/10.1016/s1074-7613(00)80454-9)

11. [Dorina A, Danielle C. The multifaceted roles of Bcl11b in thymic and peripheral T cells - impact on immune diseases. J Immunol. 2014;193: 2059.](http://paperpile.com/b/dsazkK/KGzv6)

12. [Ho I-C, Tai T-S, Pai S-Y. GATA3 and the T-cell lineage: essential functions before and after T-helper-2-cell differentiation. Nat Rev Immunol. 2009;9: 125.](http://paperpile.com/b/dsazkK/YHyxG)

13. [Zhang X, Lu Y, Cao X, Zhen T, Kovalovsky D. Zbtb1 prevents default myeloid differentiation of lymphoid-primed multipotent progenitors. Oncotarget. 2016;7: 58768.](http://paperpile.com/b/dsazkK/3Rs4Q)

14. [Maeda T. Regulation of hematopoietic development by ZBTB transcription factors. Int J Hematol. 2016;104: 310.](http://paperpile.com/b/dsazkK/ZagIM)

15. [Mallory MJ, Jackson J, Weber B, Chi A, Heyd F, Lynch KW. Signal- and Development-Dependent Alternative Splicing of LEF1 in T Cells Is Controlled by CELF2. Mol Cell Biol. 2011;31: 2184–2195.](http://paperpile.com/b/dsazkK/0kive)

16. [Burchill MA, Goetz CA, Prlic M, O’Neil JJ, Harmon IR, Bensinger SJ, et al. Distinct effects of STAT5 activation on CD4+ and CD8+ T cell homeostasis: development of CD4+CD25+ regulatory T cells versus CD8+ memory T cells. J Immunol. 2003;171. doi:](http://paperpile.com/b/dsazkK/YwDPc)[10.4049/jimmunol.171.11.5853](http://dx.doi.org/10.4049/jimmunol.171.11.5853)

17. [David L. Owen MAF. STAT5 and CD4 + T Cell Immunity. F1000Res. 2017;6. doi:](http://paperpile.com/b/dsazkK/MvQ8k)[10.12688/f1000research.9838.1](http://dx.doi.org/10.12688/f1000research.9838.1)

18. [Wang C, Liu X, Liu Y, Zhang Q, Yao Z, Huang B, et al. Zinc finger protein 64 promotes Toll-like receptor-triggered proinflammatory and type I interferon production in macrophages by enhancing p65 subunit activation. J Biol Chem. 2013;288. doi:](http://paperpile.com/b/dsazkK/N7rpO)[10.1074/jbc.M113.473397](http://dx.doi.org/10.1074/jbc.M113.473397)

19. [A Transcription Factor Addiction in Leukemia Imposed by the MLL Promoter Sequence. Cancer Cell. 2018;34: 970–981.e8.](http://paperpile.com/b/dsazkK/tj2FL)

20. [Wei L, Laurence A, O’Shea JJ. New Insights into the Roles of Stat5a/b and Stat3 in T Cell Development and Differentiation. Semin Cell Dev Biol. 2008;19: 394.](http://paperpile.com/b/dsazkK/jhkPw)

21. [O’Shea JJ, Lahesmaa R, Vahedi G, Laurence A, Kanno Y. Genomic views of STAT function in CD4+ T helper cell differentiation: new technology brings new insights and new questions. Nat Rev Immunol. 2011;11: 239.](http://paperpile.com/b/dsazkK/hHYFZ)

22. [Abboud G, Stanfield J, Tahiliani V, Desai P, Hutchinson TE, Lorentsen KJ, et al. Transcription Factor Bcl11b Controls Effector and Memory CD8 T cell Fate Decision and Function during Poxvirus Infection. Front Immunol. 2016;7. doi:](http://paperpile.com/b/dsazkK/tddix)[10.3389/fimmu.2016.00425](http://dx.doi.org/10.3389/fimmu.2016.00425)

23. [Kanhere A, Hertweck A, Bhatia U, Refik Gökmen M, Perucha E, Jackson I, et al. T-bet and GATA3 orchestrate Th1 and Th2 differentiation through lineage-specific targeting of distal regulatory elements. Nat Commun. 2012;3: 1–12.](http://paperpile.com/b/dsazkK/H4dMZ)

24. [Joshi NS, Cui W, Dominguez CX, Chen JH, Hand TW, Kaech SM. Increased Numbers of Preexisting Memory CD8 T Cells and Decreased T-bet Expression Can Restrain Terminal Differentiation of Secondary Effector and Memory CD8 T Cells. The Journal of Immunology. 2011;187: 4068–4076.](http://paperpile.com/b/dsazkK/GiJQO)

25. [Wang Y, Misumi I, Gu A-D, Anthony Curtis T, Su L, Whitmire JK, et al. GATA-3 controls T cell maintenance and proliferation downstream of TCR and cytokine signals. Nat Immunol. 2013;14: 714.](http://paperpile.com/b/dsazkK/MAiJD)

26. [Pioli PD, Whiteside SK, Weis JJ, Weis JH. Snai2 and Snai3 transcriptionally regulate cellular fitness and functionality of T cell lineages through distinct gene programs. Immunobiology. 2016;221. doi:](http://paperpile.com/b/dsazkK/g7qJf)[10.1016/j.imbio.2016.01.007](http://dx.doi.org/10.1016/j.imbio.2016.01.007)

27. [Xinyuan Z, Haihui X. Generation of memory precursors and functional memory CD8+ T cells depends on TCF-1 and LEF-1. J Immunol. 2012;189: 2722.](http://paperpile.com/b/dsazkK/o6T8D)

28. [Quintana FJ, Jin H, Burns EJ, Nadeau M, Yeste A, Kumar D, et al. Aiolos promotes TH17 differentiation by directly silencing Il2 expression. Nat Immunol. 13: 770.](http://paperpile.com/b/dsazkK/l3Aiv)

29. [Burrows K, Antignano F, Bramhall M, Chenery A, Scheer S, Korinek V, et al. The transcriptional repressor HIC1 regulates intestinal immune homeostasis. Mucosal Immunol. 2017;10: 1518–1528.](http://paperpile.com/b/dsazkK/N7Zfq)

30. [Kurachi M, Anthony Barnitz R, Yosef N, Odorizzi PM, DiIorio MA, Lemieux ME, et al. The transcription factor BATF operates as an essential differentiation checkpoint in early effector CD8 + T cells. Nat Immunol. 2014;15: 373–383.](http://paperpile.com/b/dsazkK/yp5GZ)

31. [Li J, He Y, Hao J, Ni L, Dong C. High Levels of Eomes Promote Exhaustion of Anti-tumor CD8+ T Cells. Front Immunol. 2018;9. doi:](http://paperpile.com/b/dsazkK/MWRFb)[10.3389/fimmu.2018.02981](http://dx.doi.org/10.3389/fimmu.2018.02981)

32. [Melichar HJ, Narayan K, Der SD, Hiraoka Y, Gardiol N, Jeannet G, et al. Regulation of gammadelta versus alphabeta T lymphocyte differentiation by the transcription factor SOX13. Science. 2007;315. doi:](http://paperpile.com/b/dsazkK/igFzp)[10.1126/science.1135344](http://dx.doi.org/10.1126/science.1135344)

33. [Hatano S, Murakami T, Noguchi N, Yamada H, Yoshikai Y. CD5 − NK1.1 γδ T Cells that Develop in a Bcl11b-Independent Manner Participate in Early Protection against Infection. Cell Reports. 2017. pp. 1191–1202. doi:](http://paperpile.com/b/dsazkK/zMC0A)[10.1016/j.celrep.2017.10.007](http://dx.doi.org/10.1016/j.celrep.2017.10.007)

34. [Rodríguez-Gómez IM, Talker SC, Käser T, Stadler M, Reiter L, Ladinig A, et al. Expression of T-Bet, Eomesodermin, and GATA-3 Correlates With Distinct Phenotypes and Functional Properties in Porcine γδ T Cells. Front Immunol. 2019;10: 396.](http://paperpile.com/b/dsazkK/7r6I9)

35. [Peters C, Häsler R, Wesch D, Kabelitz D. Human Vδ2 T cells are a major source of interleukin-9. Proc Natl Acad Sci U S A. 2016;113: 12520–12525.](http://paperpile.com/b/dsazkK/yHr8y)

36. [Seiler MP, Mathew R, Liszewski MK, Spooner CJ, Barr K, Meng F, et al. Elevated and sustained expression of the transcription factors Egr1 and Egr2 controls NKT lineage differentiation in response to TCR signaling. Nat Immunol. 2012;13: 264–271.](http://paperpile.com/b/dsazkK/BhkxJ)

37. [Zhang B, Jiao A, Dai M, Wiest DL, Zhuang Y. Id3 Restricts γδ NKT Cell Expansion by Controlling Egr2 and c-Myc Activity. The Journal of Immunology. 2018;201: 1452–1459.](http://paperpile.com/b/dsazkK/sbrzj)

38. [Morgan E. Parker MC. Regulation of γδ T Cell Effector Diversification in the Thymus. Front Immunol. 2020;11. doi:](http://paperpile.com/b/dsazkK/YeSKV)[10.3389/fimmu.2020.00042](http://dx.doi.org/10.3389/fimmu.2020.00042)

39. [Li K, Wu Y, Li Y, Yu Q, Tian Z, Wei H, et al. Landscape and dynamics of the transcriptional regulatory network during natural killer cell differentiation. doi:](http://paperpile.com/b/dsazkK/vXNYK)[10.1101/572768](http://dx.doi.org/10.1101/572768)

40. [Levanon D, Negreanu V, Lotem J, Bone KR, Brenner O, Leshkowitz D, et al. Transcription Factor Runx3 Regulates Interleukin-15-Dependent Natural Killer Cell Activation. Mol Cell Biol. 2014;34: 1158.](http://paperpile.com/b/dsazkK/MWPJA)

41. [Kanda M, Yamanaka H, Kojo S, Usui Y, Honda H, Sotomaru Y, et al. Transcriptional regulator Bhlhe40 works as a cofactor of T-bet in the regulation of IFN-γ production in iNKT cells. Proc Natl Acad Sci U S A. 2016;113: E3394–E3402.](http://paperpile.com/b/dsazkK/tuY5f)

42. [Samson SI, Richard O, Tavian M, Ranson T, Vosshenrich CA, Colucci F, et al. GATA-3 promotes maturation, IFN-gamma production, and liver-specific homing of NK cells. Immunity. 2003;19. doi:](http://paperpile.com/b/dsazkK/CH2Li)[10.1016/s1074-7613(03)00294-2](http://dx.doi.org/10.1016/s1074-7613(03)00294-2)

43. [Firth MA, Madera S, Beaulieu AM, Gasteiger G, Castillo EF, Schluns KS, et al. Nfil3-independent lineage maintenance and antiviral response of natural killer cells. J Exp Med. 2013;210: 2981.](http://paperpile.com/b/dsazkK/i0v0P)

44. [Jamil KM, Hydes TJ, Cheent KS, Cassidy SA, Traherne JA, Jayaraman J, et al. STAT4-associated natural killer cell tolerance following liver transplantation. Gut. 2017;66. doi:](http://paperpile.com/b/dsazkK/DPX7h)[10.1136/gutjnl-2015-309395](http://dx.doi.org/10.1136/gutjnl-2015-309395)
