# Supplementary material for: Systems-level identification of key transcription factors in immune cell specification
Source: PLoS Comput Biol. 2022 Sep 26;18(9):e1010116. doi: 10.1371/journal.pcbi.1010116 (PMC9536753; doi:10.1371/journal.pcbi.1010116)
Supplement: S5 Table — (DOCX) [file pcbi.1010116.s012.docx]

#### **Table S5. Predicted putative driver TFs in memory cells and tissue resident cells**

Memory cell-specific TFs

| Predicted TFs | p-value | log2 fold change | Evidence |
| --- | --- | --- | --- |
| *Osr2* | *6.71E-8* | *0.492* | Unknown |
| *Zfp777* | *3.69E-6* | *0.713* | Unknown |
| *Foxo1* | *3.15E-5* | *0.741* | Directs and maintains memory T cell differentiation and function[[1–7]](https://paperpile.com/c/MsKXAB/69Ra+t95T+PxKt+Iy8n+VUfH+DtXm+UYjq) |
| *Ikzf3* | *1.27E-4* | *1.139* | Relates to memory T cell function[[8–10]](https://paperpile.com/c/MsKXAB/rDNJM+RK2LF+SCIZj) |
| *Foxp1* | *7.35E-4* | *0.707* | Expression downregulated in memory cells[[11–18]](https://paperpile.com/c/MsKXAB/fWYBg+1abaA+VCAjY+ENbJ7+yPG3M+oBB7O+qsV56+3K0nI) |
| *Zfp3* | *1.62E-3* | *0.484* | Unknown |
| *Prdm9* | *1.71E-3* | *0.855* | Unknown |
| *Tbx2* | *1.71E-3* | *0.232* | Unknown |
| *Creb2* | *2.79E-3* | *1.588* | Relates to the establishment of memory T cells[[19,20]](https://paperpile.com/c/MsKXAB/QMe1U+bfArs) |
| *Pbx1* | *5.16E-3* | *0.415* | Relates to T cell development and differentiation[[21–24]](https://paperpile.com/c/MsKXAB/gOlTW+KL4BE+JTdXT+qhsvY) |
| *Klf2* | *5.26E-3* | *1.188* | Involved in T cell trafficking and memory formation[[25–27]](https://paperpile.com/c/MsKXAB/B9w73+EXqKC+X5Coa) |
| *Ahctf1* | *1.03E-2* | *0.284* | Unknown |
| *Ets1* | *1.13E-2* | *0.825* | Highly expressed in memory B cells[[28,29]](https://paperpile.com/c/MsKXAB/5VaCk+EERVR) |
| *Lcorl* | *1.55E-2* | *0.317* | Unknown |
| *Pou6f1* | *2.03E-2* | *1.337* | Upregulated in innate memory and memory CD8^+^ T cells [[30,31]](https://paperpile.com/c/MsKXAB/VbLvt+QqvHr) |
| *Pou4f1* | *2.10E-2* | *1.040* | Unknown |
| *Crx* | *2.41E-2* | *0.181* | Unknown |
| *Zfp143* | *2.86E-2* | *0.320* | Gene highly accessibility in T cell in a terminally dysfunctional state[[32]](https://paperpile.com/c/MsKXAB/w4V2x) |
| *Ctcf* | *3.76E-2* | *0.164* | Association with T cell differentiation gene programs[[33]](https://paperpile.com/c/MsKXAB/HKVyA) |
| *Cphx1* | *3.81E-2* | *1.224* | Unknown |
| *Hsf4* | *4.84E-2* | *0.277* | Unknown |
| *Prox2* | *4.91E-2* | *0.855* | Unknown |
| *Zfp324* | *5.22E-2* | *0.662* | Unknown |
| *Hic1* | *6.61E-2* | *0.316* | Promotes intestinal T cell residency[[34]](https://paperpile.com/c/MsKXAB/NZigZ) |
| *Zfp300* | *6.79E-2* | *0.855* | Unknown |
| *Zfp90* | *6.85E-2* | *0.194* | Unknown |
| *Scrt1* | *8.43E-2* | *0.602* | Unknown |
| *Hsf1* | *8.88E-2* | *0.429* | Unknown |
| *Zbtb22* | *9.14E-2* | *0.597* | Unknown |
| *Elf1* | *9.54E-2* | *0.244* | Unknown |

####

Tissue-residency-specific TFs

| Predicted TFs | p-value | log2 fold change | Evidence |
| --- | --- | --- | --- |
| *Hic1* | *3.54E-5* | *1.543* | Regulation of T cell residency and function in the intestine[[34,35]](https://paperpile.com/c/MsKXAB/0w8xu+NZigZ) |
| *Ahr* | *3.91E-5* | *2.636* | Required for long term persistence of T_RM_ in the epidermis[[36]](https://paperpile.com/c/MsKXAB/x9tfA) |
| *Atf3* | *3.93E-5* | *3.175* | Enriched expression in T_RM_ cells[[37,38]](https://paperpile.com/c/MsKXAB/Kgaic+TNKrn) |
| *Relb* | *8.05E-5* | *1.491* | Unknown |
| *Rbpj* | *1.40E-4* | *0.897* | Required for T_RM_ development in the lungs[[37]](https://paperpile.com/c/MsKXAB/Kgaic) |
| *Crem* | *9.85E-4* | *1.564* | Enriched in interstitial lung T_RM_ cells[[39]](https://paperpile.com/c/MsKXAB/4XwUe) |
| *Nfkb1* | *1.25E-3* | *1.479* | Enriched expression in T_RM_ cells[[40]](https://paperpile.com/c/MsKXAB/3HOML) |
| *Rora* | *2.31E-3* | *2.052* | Enriched in distinct T_RM_ populations[[40–43]](https://paperpile.com/c/MsKXAB/f3SwX+3HOML+EYkLq+wa35p) |
| *Maf* | *3.76E-3* | *2.387* | Promotes tissue-residency program in Th17 cells[[44]](https://paperpile.com/c/MsKXAB/LuP94) |
| *Rorc* | *7.21E-3* | *4.435* | Unknown |
| *Nfil3* | *7.64E-3* | *1.019* | Relate to NK cells and ILC1 development[[45–48]](https://paperpile.com/c/MsKXAB/AmgWl+l2Bew+VfaXN+SPSo8) |
| *Kdm2b* | *8.19E-3* | *1.544* | Unknown |
| *Hey1* | *8.86E-3* | *1.564* | NOTCH target genes[[49]](https://paperpile.com/c/MsKXAB/QcVz6) |
| *Npas2* | *1.05E-2* | *1.472* | Unknown |
| *Snai1* | *1.66E-2* | *1.021* | Unknown |
| *Mafg* | *1.67E-2* | *0.841* | Regulates Th17 responses in the intestine[[50]](https://paperpile.com/c/MsKXAB/O0Pzv) |
| *Epas1* | *1.84E-2* | *2.082* | Elevated expression in lung T_RM_ cells[[37,51]](https://paperpile.com/c/MsKXAB/fvXe0+Kgaic) |
| *Gata5* | *1.90E-2* | *1.169* | Unknown |
| *Hnf4g* | *1.96E-2* | *1.087* | Unknown |
| *Nfatc2* | *2.20E-2* | *0.990* | Induce CD103 expression in CD8^+^ T cells[[52]](https://paperpile.com/c/MsKXAB/xTgcf) |
| *Jund* | *2.34E-2* | *0.689* | Important in regulating tissue-specific Tregs[[53,54]](https://paperpile.com/c/MsKXAB/ILYZ5+02jJC) |
| *Srebf2* | *3.01E-2* | *0.507* | Unknown |
| *Mycn* | *3.14E-2* | *1.989* | Unknown |
| *Nkx6-2* | *3.40E-2* | *1.243* | Unknown |
| *Etv5* | *3.59E-2* | *0.975* | CIC target gene responsible for regulation of CD8^+^ T_RM_ cells[[55,56]](https://paperpile.com/c/MsKXAB/9b0ts+BtU9X) |
| *Vdr* | *3.70E-2* | *1.172* | Regulate MAIT cell frequency and function[[57]](https://paperpile.com/c/MsKXAB/ue8f2)  Role in T cell development, function and homing[[58]](https://paperpile.com/c/MsKXAB/jmsmn)  Regulate tissue resident macrophage response[[59]](https://paperpile.com/c/MsKXAB/T2aOy) |
| *Mitf* | *3.74E-2* | *1.334* | Unknown |
| *Nr1d1* | *4.29E-2* | *2.564* | Regulates Th17 development and associated autoimmunity[[60]](https://paperpile.com/c/MsKXAB/vR8Xv) |
| *Zik1* | *4.31E-2* | *0.944* | Unknown |
| *Nr1h3* | *4.44E-2* | *0.952* | Role in generation of tissue-specific macrophage populations[[61–64]](https://paperpile.com/c/MsKXAB/bljVZ+fByZ5+OZGiP+0pVPp) |
| *Creb3l2* | *4.49E-2* | *0.646* | Unknown |
| *Zfp523* | *5.01E-2* | *0.648* | Unknown |
| *Klf9* | *5.07E-2* | *0.949* | Unknown |
| *Maff* | *5.64E-2* | *1.459* | Expressed in tissue ILCs and adaptive-like NK cells |
| *Ppard* | *5.73E-2* | *0.560* | Expression upregulated in pancreas T_RM_[_[65]_](https://paperpile.com/c/MsKXAB/dPEVA) |
| *Gata3* | *5.79E-2* | *1.010* | Functions in T cell and ILC differentiation[[66,67]](https://paperpile.com/c/MsKXAB/NLzxT+LuBS0) |
| *Foxs1* | *5.85E-2* | *2.178* | Unknown |
| *Cebpb* | *6.22E-2* | *0.833* | Role in generation of tissue-resident macrophage populationss[[68]](https://paperpile.com/c/MsKXAB/iNfU6) |
| *Heyl* | *6.37E-2* | *1.080* | Unknown |
| *Lbx2* | *7.13E-2* | *2.250* | Unknown |
| *Cebpd* | *7.27E-2* | *1.441* | Regulates MAIT cell trafficking[[69]](https://paperpile.com/c/MsKXAB/jVh2S) |
| *Gmeb2* | *7.44E-2* | *0.538* | Unknown |
| *Hes1* | *8.39E-2* | *1.956* | Increased expression in colonic macrophages[[70]](https://paperpile.com/c/MsKXAB/gw10S) |
| *Dlx4* | *9.12E-2* | *0.571* | Unknown |
| *Pparg* | *9.18E-2* | *1.377* | Directs accumulation and phenotype of adipose tissue Tregs[[71]](https://paperpile.com/c/MsKXAB/5SA3p) |
| *Zfp41* | *9.21E-2* | *0.629* | Unknown |
| *Rax* | *9.72E-2* | *1.185* | Unknown |
| *Tcf4* | *9.93E-2* | *0.764* | Unknown |
| *Arid3a* | *1.06E-2* | *0.685* | Unknown |
| *Hsf2* | *1.19E-2* | *0.534* | Unknown |
| *Zbtb48* | *1.19E-2* | *0.505* | Unknown |
| *Tet3* | *1.21E-2* | *0.824* | Unknown |
| *Scrt1* | *1.23E-2* | *0.950* | Unknown |

####

#### Reference

1. [Utzschneider DT, Delpoux A, Wieland D, Huang X, Lai CY, Hofmann M, et al. Active Maintenance of T Cell Memory in Acute and Chronic Viral Infection Depends on Continuous Expression of FOXO1. Cell Rep. 2018;22. doi:](http://paperpile.com/b/MsKXAB/69Ra)[10.1016/j.celrep.2018.03.020](http://dx.doi.org/10.1016/j.celrep.2018.03.020)

2. [Rao RR, Li Q, Mr GB, Shrikant PA. Transcription factor Foxo1 represses T-bet-mediated effector functions and promotes memory CD8(+) T cell differentiation. Immunity. 2012;36. doi:](http://paperpile.com/b/MsKXAB/t95T)[10.1016/j.immuni.2012.01.015](http://dx.doi.org/10.1016/j.immuni.2012.01.015)

3. [Kim MV, Ouyang W, Liao W, Zhang MQ, Li MO. The transcription factor Foxo1 controls central-memory CD8+ T cell responses to infection. Immunity. 2013;39. doi:](http://paperpile.com/b/MsKXAB/PxKt)[10.1016/j.immuni.2013.07.013](http://dx.doi.org/10.1016/j.immuni.2013.07.013)

4. [Hess MR, Doedens AL, Goldrath AW, Hedrick SM. Differentiation of CD8 memory T cells depends on Foxo1. J Exp Med. 2013;210. doi:](http://paperpile.com/b/MsKXAB/Iy8n)[10.1084/jem.20130392](http://dx.doi.org/10.1084/jem.20130392)

5. [Delpoux A, Michelini RH, Verma S, Lai CY, Omilusik KD, Utzschneider DT, et al. Continuous activity of Foxo1 is required to prevent anergy and maintain the memory state of CD8 + T cells. J Exp Med. 2018;215. doi:](http://paperpile.com/b/MsKXAB/VUfH)[10.1084/jem.20170697](http://dx.doi.org/10.1084/jem.20170697)

6. [Delpoux A, Lai CY, Hedrick SM, Doedens AL. FOXO1 opposition of CD8 + T cell effector programming confers early memory properties and phenotypic diversity. Proc Natl Acad Sci U S A. 2017;114. doi:](http://paperpile.com/b/MsKXAB/DtXm)[10.1073/pnas.1618916114](http://dx.doi.org/10.1073/pnas.1618916114)

7. [Gray SM, Amezquita RA, Guan T, Kleinstein SH, Kaech SM. Polycomb Repressive Complex 2-Mediated Chromatin Repression Guides Effector CD8 + T Cell Terminal Differentiation and Loss of Multipotency. Immunity. 2017;46. doi:](http://paperpile.com/b/MsKXAB/UYjq)[10.1016/j.immuni.2017.03.012](http://dx.doi.org/10.1016/j.immuni.2017.03.012)

8. [Clambey ET, Collins B, Young MH, Eberlein J, David A, Kappler JW, et al. The Ikaros Transcription Factor Regulates Responsiveness to IL-12 and Expression of IL-2 Receptor Alpha in Mature, Activated CD8 T Cells. PLoS One. 2013;8: e57435.](http://paperpile.com/b/MsKXAB/rDNJM)

9. [Quintana FJ, Jin H, Burns EJ, Nadeau M, Yeste A, Kumar D, et al. Aiolos promotes TH17 differentiation by directly silencing Il2 expression. Nat Immunol. 13: 770.](http://paperpile.com/b/MsKXAB/RK2LF)

10. [Powell MD, Read KA, Sreekumar BK, Oestreich KJ. Ikaros Zinc Finger Transcription Factors: Regulators of Cytokine Signaling Pathways and CD4+ T Helper Cell Differentiation. Front Immunol. 2019;10. doi:](http://paperpile.com/b/MsKXAB/SCIZj)[10.3389/fimmu.2019.01299](http://dx.doi.org/10.3389/fimmu.2019.01299)

11. [Garaud S, Roufosse F, De Silva P, Gu-Trantien C, Lodewyckx J-N, Duvillier H, et al. FOXP1 is a regulator of quiescence in healthy human CD4 T cells and is constitutively repressed in T cells from patients with lymphoproliferative disorders. European Journal of Immunology. 2017. pp. 168–179. doi:](http://paperpile.com/b/MsKXAB/fWYBg)[10.1002/eji.201646373](http://dx.doi.org/10.1002/eji.201646373)

12. [Patzelt T, Keppler SJ, Gorka O, Thoene S, Wartewig T, Reth M, et al. Foxp1 controls mature B cell survival and the development of follicular and B-1 B cells. Proc Natl Acad Sci U S A. 2018;115: 3120–3125.](http://paperpile.com/b/MsKXAB/1abaA)

13. [Wei H, Geng J, Shi B, Liu Z, Wang Y-H, Stevens AC, et al. Cutting Edge: Foxp1 Controls Naive CD8+ T Cell Quiescence by Simultaneously Repressing Key Pathways in Cellular Metabolism and Cell Cycle Progression. The Journal of Immunology. 2016;196: 3537–3541.](http://paperpile.com/b/MsKXAB/VCAjY)

14. [Feng X, Wang H, Takata H, Day TJ, Willen J, Hu H. Transcription factor Foxp1 exerts essential cell-intrinsic regulation of the quiescence of naive T cells. Nat Immunol. 2011;12. doi:](http://paperpile.com/b/MsKXAB/ENbJ7)[10.1038/ni.2034](http://dx.doi.org/10.1038/ni.2034)

15. [Wang H, Geng J, Wen X, Bi E, Kossenkov AV, Wolf AI, et al. The transcription factor Foxp1 is a critical negative regulator of the differentiation of follicular helper T cells. Nat Immunol. 2014;15: 667–675.](http://paperpile.com/b/MsKXAB/yPG3M)

16. [Hu H, Wang B, Borde M, Nardone J, Maika S, Allred L, et al. Foxp1 is an essential transcriptional regulator of B cell development. Nat Immunol. 2006;7: 819–826.](http://paperpile.com/b/MsKXAB/oBB7O)

17. [Konopacki C, Pritykin Y, Rubtsov Y, Leslie CS, Rudensky AY. Transcription factor Foxp1 regulates Foxp3 chromatin binding and coordinates regulatory T cell function. Nat Immunol. 2019;20: 232–242.](http://paperpile.com/b/MsKXAB/qsV56)

18. [Ren J, Han L, Tang J, Liu Y, Deng X, Liu Q, et al. Foxp1 is critical for the maintenance of regulatory T-cell homeostasis and suppressive function. PLoS Biol. 2019;17. doi:](http://paperpile.com/b/MsKXAB/3K0nI)[10.1371/journal.pbio.3000270](http://dx.doi.org/10.1371/journal.pbio.3000270)

19. [Rutishauser RL, Kaech SM. Generating diversity: transcriptional regulation of effector and memory CD8 T-cell differentiation. Immunol Rev. 2010;235: 219–233.](http://paperpile.com/b/MsKXAB/QMe1U)

20. [Maekawa Y, Minato Y, Ishifune C, Kurihara T, Kitamura A, Kojima H, et al. Notch2 integrates signaling by the transcription factors RBP-J and CREB1 to promote T cell cytotoxicity. Nat Immunol. 2008;9: 1140–1147.](http://paperpile.com/b/MsKXAB/bfArs)

21. [The PBX1 lupus susceptibility gene regulates CD44 expression. Mol Immunol. 2017;85: 148–154.](http://paperpile.com/b/MsKXAB/gOlTW)

22. [Choi S-C, Hutchinson TE, Titov AA, Seay HR, Li S, Brusko TM, et al. The Lupus Susceptibility Gene Pbx1 Regulates the Balance between Follicular Helper T Cell and Regulatory T Cell Differentiation. The Journal of Immunology. 2016;197: 458–469.](http://paperpile.com/b/MsKXAB/KL4BE)

23. [Sanyal M, Tung JW, Dejbakhsh-Jones S, Strober S, Herzenberg LA, Cleary ML. The Transcription Factor Pbx1 Is Required for the Development of Double Positive Thymic T Cells. Blood. 2004;104: 2771–2771.](http://paperpile.com/b/MsKXAB/JTdXT)

24. [B-cell development fails in the absence of the Pbx1 proto-oncogene. Blood. 2007;109: 4191–4199.](http://paperpile.com/b/MsKXAB/qhsvY)

25. [Skon C, Lee J-Y, Jameson S. KLF2 and S1Pr1: Aiding in memory T cell trafficking and retention in non-lymphoid tissue (173.16). The Journal of Immunology. 2012;188: 173.16–173.16.](http://paperpile.com/b/MsKXAB/B9w73)

26. [Laidlaw BJ, Duan L, Xu Y, Vazquez SE, Cyster JG. The transcription factor Hhex cooperates with the corepressor Tle3 to promote memory B cell development. Nat Immunol. 2020;21: 1082–1093.](http://paperpile.com/b/MsKXAB/EXqKC)

27. [Bhattacharya D, Cheah MT, Franco CB, Hosen N, Pin CL, Sha WC, et al. Transcriptional profiling of antigen-dependent murine B cell differentiation and memory formation. J Immunol. 2007;179. doi:](http://paperpile.com/b/MsKXAB/X5Coa)[10.4049/jimmunol.179.10.6808](http://dx.doi.org/10.4049/jimmunol.179.10.6808)

28. [Garrett-Sinha LA. Review of Ets1 structure, function, and roles in immunity. Cell Mol Life Sci. 2013;70: 3375.](http://paperpile.com/b/MsKXAB/5VaCk)

29. [Grenningloh R, Tai T-S, Frahm N, Hongo TC, Chicoine AT, Brander C, et al. Ets-1 Maintains IL-7 Receptor Expression in Peripheral T Cells. The Journal of Immunology. 2011;186: 969–976.](http://paperpile.com/b/MsKXAB/EERVR)

30. [Istaces N, Splittgerber M, Silva VL, Nguyen M, Thomas S, Le A, et al. EOMES interacts with RUNX3 and BRG1 to promote innate memory cell formation through epigenetic reprogramming. Nat Commun. 2019;10: 1–17.](http://paperpile.com/b/MsKXAB/VbLvt)

31. [Yu B, Zhang K, Milner JJ, Toma C, Chen R, Scott-Browne JP, et al. Epigenetic landscapes reveal transcription factors that regulate CD8 T cell differentiation. Nat Immunol. 2017;18: 573–582.](http://paperpile.com/b/MsKXAB/QqvHr)

32. [Pritykin Y, van der Veeken J, Pine AR, Zhong Y, Sahin M, Mazutis L, et al. A unified atlas of CD8 T cell dysfunctional states in cancer and infection. Mol Cell. 2021. doi:](http://paperpile.com/b/MsKXAB/w4V2x)[10.1016/j.molcel.2021.03.045](http://dx.doi.org/10.1016/j.molcel.2021.03.045)

33. [CCCTC-Binding Factor Translates Interleukin 2- and α-Ketoglutarate-Sensitive Metabolic Changes in T Cells into Context-Dependent Gene Programs. Immunity. 2017;47: 251–267.e7.](http://paperpile.com/b/MsKXAB/HKVyA)

34. [Bramhall M, Rodrigues G, Christo S, Mackay L, Zaph C. T cell-intrinsic expression of HIC1 links retinoic acid to tissue residency. The Journal of Immunology. 2020;204: 155.3–155.3.](http://paperpile.com/b/MsKXAB/NZigZ)

35. [Burrows K, Antignano F, Bramhall M, Chenery A, Scheer S, Korinek V, et al. The transcriptional repressor HIC1 regulates intestinal immune homeostasis. Mucosal Immunol. 2017;10: 1518–1528.](http://paperpile.com/b/MsKXAB/0w8xu)

36. [Zaid A, Mackay LK, Rahimpour A, Braun A, Veldhoen M, Carbone FR, et al. Persistence of skin-resident memory T cells within an epidermal niche. Proc Natl Acad Sci U S A. 2014;111: 5307–5312.](http://paperpile.com/b/MsKXAB/x9tfA)

37. [Hombrink P, Helbig C, Backer RA, Piet B, Oja AE, Stark R, et al. Programs for the persistence, vigilance and control of human CD8 + lung-resident memory T cells. Nat Immunol. 2016;17: 1467–1478.](http://paperpile.com/b/MsKXAB/Kgaic)

38. [Li J, Olshansky M, Carbone FR, Ma JZ. Transcriptional Analysis of T Cells Resident in Human Skin. PLoS One. 2016;11. doi:](http://paperpile.com/b/MsKXAB/TNKrn)[10.1371/journal.pone.0148351](http://dx.doi.org/10.1371/journal.pone.0148351)

39. [Hayward SL, Scharer CD, Cartwright EK, Takamura S, Li Z-RT, Boss JM, et al. Environmental cues regulate epigenetic reprogramming of airway-resident memory CD8 + T cells. Nat Immunol. 2020;21: 309–320.](http://paperpile.com/b/MsKXAB/4XwUe)

40. [Kurd NS, He Z, Louis TL, Justin Milner J, Omilusik KD, Jin W, et al. Early precursors and molecular determinants of tissue-resident memory CD8+ T lymphocytes revealed by single-cell RNA sequencing. Science Immunology. 2020;5. doi:](http://paperpile.com/b/MsKXAB/3HOML)[10.1126/sciimmunol.aaz6894](http://dx.doi.org/10.1126/sciimmunol.aaz6894)

41. [Miragaia RJ, Gomes T, Chomka A, Jardine L, Riedel A, Hegazy AN, et al. Single-Cell Transcriptomics of Regulatory T Cells Reveals Trajectories of Tissue Adaptation. Immunity. 2019;50: 493–504.e7.](http://paperpile.com/b/MsKXAB/f3SwX)

42. [Malhotra N, Leyva-Castillo JM, Jadhav U, Barreiro O, Kam C, O’Neill NK, et al. RORα-expressing T regulatory cells restrain allergic skin inflammation. Science immunology. 2018;3. doi:](http://paperpile.com/b/MsKXAB/EYkLq)[10.1126/sciimmunol.aao6923](http://dx.doi.org/10.1126/sciimmunol.aao6923)

43. [Vaeth M, Wang Y-H, Eckstein M, Yang J, Silverman GJ, Lacruz RS, et al. Tissue resident and follicular Treg cell differentiation is regulated by CRAC channels. Nat Commun. 2019;10: 1–16.](http://paperpile.com/b/MsKXAB/wa35p)

44. [Aschenbrenner D, Foglierini M, Jarrossay D, Hu D, Weiner HL, Kuchroo VK, et al. Immunoregulatory and tissue-residency programs modulated by c-MAF in human TH17 cells. Nat Immunol. 2018;19: 1126.](http://paperpile.com/b/MsKXAB/LuP94)

45. [Erick TK, Anderson CK, Reilly EC, Wands JR, Brossay L. NFIL3 Expression Distinguishes Tissue-Resident NK Cells and Conventional NK-like Cells in the Mouse Submandibular Glands. The Journal of Immunology. 2016;197: 2485–2491.](http://paperpile.com/b/MsKXAB/AmgWl)

46. [Elaheh Hashemi SM. Tissue-Resident NK Cells: Development, Maturation, and Clinical Relevance. Cancers . 2020;12. doi:](http://paperpile.com/b/MsKXAB/l2Bew)[10.3390/cancers12061553](http://dx.doi.org/10.3390/cancers12061553)

47. [Dadi S, Li MO. Tissue-resident lymphocytes: sentinel of the transformed tissue. Journal for ImmunoTherapy of Cancer. 2017;5: 1–3.](http://paperpile.com/b/MsKXAB/VfaXN)

48. [Cortez VS, Fuchs A, Cella M, Gilfillan S, Colonna M. Cutting Edge: Salivary Gland NK Cells Develop Independently of Nfil3 in Steady-State. The Journal of Immunology. 2014;192: 4487–4491.](http://paperpile.com/b/MsKXAB/SPSo8)

49. [Michelle A. K, Justine E. R. NOTCH Signaling in T-Cell-Mediated Anti-Tumor Immunity and T-Cell-Based Immunotherapies. Front Immunol. 2018;9. doi:](http://paperpile.com/b/MsKXAB/QcVz6)[10.3389/fimmu.2018.01718](http://dx.doi.org/10.3389/fimmu.2018.01718)

50. [Imbratta C, Hussein H, Andris F, Verdeil G. c-MAF, a Swiss Army Knife for Tolerance in Lymphocytes. Front Immunol. 2020;11. doi:](http://paperpile.com/b/MsKXAB/O0Pzv)[10.3389/fimmu.2020.00206](http://dx.doi.org/10.3389/fimmu.2020.00206)

51. [Liikanen I, Lauhan C, Quon S, Omilusik K, Phan AT, Bartrolí LB, et al. Hypoxia-inducible factor activity promotes antitumor effector function and tissue residency by CD8+ T cells. J Clin Invest. 2021;131. doi:](http://paperpile.com/b/MsKXAB/fvXe0)[10.1172/JCI143729](http://dx.doi.org/10.1172/JCI143729)

52. [Mokrani M ’barka, Klibi J, Bluteau D, Bismuth G, Mami-Chouaib F. Smad and NFAT pathways cooperate to induce CD103 expression in human CD8 T lymphocytes. J Immunol. 2014;192: 2471–2479.](http://paperpile.com/b/MsKXAB/xTgcf)

53. [Wheaton JD, Ciofani M. JunB Controls Intestinal Effector Programs in Regulatory T Cells. Front Immunol. 2020;11. doi:](http://paperpile.com/b/MsKXAB/ILYZ5)[10.3389/fimmu.2020.00444](http://dx.doi.org/10.3389/fimmu.2020.00444)

54. [DiSpirito JR, Zemmour D, Ramanan D, Cho J, Zilionis R, Klein AM, et al. Molecular diversification of regulatory T cells in nonlymphoid tissues. Science Immunology. 2018;3. doi:](http://paperpile.com/b/MsKXAB/02jJC)[10.1126/sciimmunol.aat5861](http://dx.doi.org/10.1126/sciimmunol.aat5861)

55. [Park S, Park J, Kim E, Lee Y. The Capicua/ETS Translocation Variant 5 Axis Regulates Liver-Resident Memory CD8 + T-Cell Development and the Pathogenesis of Liver Injury. Hepatology. 2019;70. doi:](http://paperpile.com/b/MsKXAB/9b0ts)[10.1002/hep.30594](http://dx.doi.org/10.1002/hep.30594)

56. [Wang Y, Zhang C. The Roles of Liver-Resident Lymphocytes in Liver Diseases. Front Immunol. 2019;10. doi:](http://paperpile.com/b/MsKXAB/BtU9X)[10.3389/fimmu.2019.01582](http://dx.doi.org/10.3389/fimmu.2019.01582)

57. [Amini A, Pang D, Hackstein C-P, Klenerman P. MAIT Cells in Barrier Tissues: Lessons from Immediate Neighbors. Front Immunol. 2020;11. doi:](http://paperpile.com/b/MsKXAB/ue8f2)[10.3389/fimmu.2020.584521](http://dx.doi.org/10.3389/fimmu.2020.584521)

58. [Chun RF, Liu PT, Modlin RL, Adams JS, Hewison M. Impact of vitamin D on immune function: lessons learned from genome-wide analysis. Front Physiol. 2014;5. doi:](http://paperpile.com/b/MsKXAB/jmsmn)[10.3389/fphys.2014.00151](http://dx.doi.org/10.3389/fphys.2014.00151)

59. [Song L, Papaioannou G, Zhao H, Luderer HF, Miller C, Dall’Osso C, et al. The Vitamin D Receptor Regulates Tissue Resident Macrophage Response to Injury. Endocrinology. 2016;157: 4066.](http://paperpile.com/b/MsKXAB/T2aOy)

60. [REV-ERBα Regulates TH17 Cell Development and Autoimmunity. Cell Rep. 2018;25: 3733–3749.e8.](http://paperpile.com/b/MsKXAB/vR8Xv)

61. [A-Gonzalez N, Guillen JA, Gallardo G, Diaz M, de la Rosa JV, Hernandez IH, et al. The Nuclear Receptor LXRα controls the functional specialization of splenic macrophages. Nat Immunol. 2013;14: 831.](http://paperpile.com/b/MsKXAB/bljVZ)

62. [A-Gonzalez N, Quintana JA, García-Silva S, Mazariegos M, de la Aleja AG, Nicolás-Ávila JA, et al. Phagocytosis imprints heterogeneity in tissue-resident macrophages. J Exp Med. 2017;214: 1281.](http://paperpile.com/b/MsKXAB/fByZ5)

63. [Mass E, Ballesteros I, Farlik M, Halbritter F, Günther P, Crozet L, et al. Specification of tissue-resident macrophages during organogenesis. Science. 2016;353. doi:](http://paperpile.com/b/MsKXAB/OZGiP)[10.1126/science.aaf4238](http://dx.doi.org/10.1126/science.aaf4238)

64. [Summers KM, Bush SJ, Hume DA. Network analysis of transcriptomic diversity amongst resident tissue macrophages and dendritic cells in the mouse mononuclear phagocyte system. PLoS Biol. 2020;18: e3000859.](http://paperpile.com/b/MsKXAB/0pVPp)

65. [Weisberg SP, Carpenter DJ, Chait M, Dogra P, Gartrell-Corrado RD, Chen AX, et al. Tissue-Resident Memory T Cells Mediate Immune Homeostasis in the Human Pancreas through the PD-1/PD-L1 Pathway. Cell Rep. 2019;29: 3916.](http://paperpile.com/b/MsKXAB/dPEVA)

66. [Fang D, Zhu J. Dynamic balance between master transcription factors determines the fates and functions of CD4 T cell and innate lymphoid cell subsets. J Exp Med. 2017;214: 1861–1876.](http://paperpile.com/b/MsKXAB/NLzxT)

67. [Zhu J. GATA3 Regulates the Development and Functions of Innate Lymphoid Cell Subsets at Multiple Stages. Front Immunol. 2017;8. doi:](http://paperpile.com/b/MsKXAB/LuBS0)[10.3389/fimmu.2017.01571](http://dx.doi.org/10.3389/fimmu.2017.01571)

68. [Cain DW, O’Koren EG, Kan MJ, Womble M, Sempowski GD, Hopper K, et al. Identification of a Tissue-Specific, C/EBPβ-Dependent Pathway of Differentiation for Murine Peritoneal Macrophages. The Journal of Immunology. 2013;191: 4665–4675.](http://paperpile.com/b/MsKXAB/iNfU6)

69. [Lee CH, Zhang HH, Singh SP, Koo L, Kabat J, Tsang H, et al. C/EBPδ drives interactions between human MAIT cells and endothelial cells that are important for extravasation. 2018 [cited 28 Jun 2021]. doi:](http://paperpile.com/b/MsKXAB/jVh2S)[10.7554/eLife.32532](http://dx.doi.org/10.7554/eLife.32532)

70. [Schridde A, Bain CC, Mayer JU, Montgomery J, Pollet E, Denecke B, et al. Tissue-specific differentiation of colonic macrophages requires TGFβ receptor-mediated signaling. Mucosal Immunol. 2017;10: 1387–1399.](http://paperpile.com/b/MsKXAB/gw10S)

71. [Cipolletta D, Feuerer M, Li A, Kamei N, Lee J, Shoelson SE, et al. PPAR-γ is a major driver of the accumulation and phenotype of adipose tissue T reg cells. Nature. 2012;486: 549–553.](http://paperpile.com/b/MsKXAB/5SA3p)
